# The Role of Danger Associated Molecular Patterns in Human Fetal Membrane Weakening

**DOI:** 10.3389/fphys.2020.00602

**Published:** 2020-06-17

**Authors:** Justin G. Padron, Chelsea A. Saito Reis, Claire E. Kendal-Wright

**Affiliations:** ^1^Anatomy, Biochemistry and Physiology, John A. Burns School of Medicine, University of Hawai‘i at Mānoa, Honolulu, HI, United States; ^2^Natural Science and Mathematics, Chaminade University of Honolulu, Honolulu, HI, United States; ^3^Obstetrics, Gynecology and Women’s Health, John A. Burns School of Medicine, University of Hawai‘i at Mānoa, Honolulu, HI, United States

**Keywords:** amnion, danger associated molecular pattern, Pattern Recognition Receptor, fetal membrane, Toll-like receptor, preterm premature rupture of fetal membrane, pathogen associated molecular pattern

## Abstract

The idea that cellular stress (including that precipitated by stretch), plays a significant role in the mechanisms initiating parturition, has gained considerable traction over the last decade. One key consequence of this cellular stress is the increased production of Danger Associated Molecular Patterns (DAMPs). This diverse family of molecules are known to initiate inflammation through their interaction with Pattern Recognition Receptors (PRRs) including, Toll-like receptors (TLRs). TLRs are the key innate immune system surveillance receptors that detect Pathogen Associated Molecular Patterns (PAMPs) during bacterial and viral infection. This is also seen during Chorioamnionitis. The activation of TLR commonly results in the activation of the pro-inflammatory transcription factor Nuclear Factor Kappa-B (NF-kB) and the downstream production of pro-inflammatory cytokines. It is thought that in the human fetal membranes both DAMPs and PAMPs are able, perhaps via their interaction with PRRs and the induction of their downstream inflammatory cascades, to lead to both tissue remodeling and weakening. Due to the high incidence of infection-driven Pre-Term Birth (PTB), including those that have preterm Premature Rupture of the Membranes (pPROM), the role of TLR in fetal membranes with Chorioamnionitis has been the subject of considerable study. Most of the work in this field has focused on the effect of PAMPs on whole pieces of fetal membrane and the resultant inflammatory cascade. This is important to understand, in order to develop novel prevention, detection, and therapeutic approaches, which aim to reduce the high number of mothers suffering from infection driven PTB, including those with pPROM. Studying the role of sterile inflammation driven by these endogenous ligands (DAMPs) activating PRRs system in the mesenchymal and epithelial cells in the amnion is important. These cells are key for the maintenance of the integrity and strength of the human fetal membranes. This review aims to (1) summarize the knowledge to date pertinent to the role of DAMPs and PRRs in fetal membrane weakening and (2) discuss the clinical potential brought by a better understanding of these pathways by pathway manipulation strategies.

## Understanding Fetal Membrane Rupture Is Important to Improve the High Rate of Preterm Birth

The human fetal membranes are an often-overlooked tissue by those studying the mechanisms of parturition. They are disregarded, as many consider that term fetal membranes are a dead tissue, or simply a membranous extension of the placenta. However, many researchers have successfully highlighted it’s importance by culturing tissue explants (Zaga et al., 2004; Astern et al., 2012) and isolated cells (Kendal-Wright et al., 2010; Sato et al., 2016), revealing its role as a complex conduit between the mother and fetus (Hadley et al., 2018). It has a large surface area for signaling and clearly contributes to the inflammation that is an established signature of parturition (Romero et al., 2007), regardless of whether it is precipitated by infection (Gomez-Lopez et al., 2018).

Parturition involves several distinct, yet integrated, physiological events; cervical ripening and dilation, contractility of the myometrium, rupture of the membranes, placental separation and uterine involution (Christiaens et al., 2008). All of these processes need to occur in a coordinated manner for the successful delivery of the fetus at term. Thus, desynchrony or the dysregulation of these events can lead to Preterm Birth (PTB) via a number of different pathways (Goldenberg et al., 2008). Approximately 20% of all preterm deliveries are by Cesarean section for maternal or fetal indications (Christiaens et al., 2008). Of the remaining cases, around a third are caused by premature preterm rupture of the membranes (pPROM), 20–25% result from intra-amniotic infection, and the remainder due to premature uterine contractions (Christiaens et al., 2008). However, approximately 60% of all preterm deliveries still remain unexplained (Christiaens et al., 2008). Epidemiological studies have suggested that preterm delivery is a condition that clusters in families (Strauss et al., 2018), and that the incidence of pPROM and the other causes of PTB differ among ethnic groups (Manuck, 2017). Although about 50% of all PTB is due to infection, antibiotics that successfully treat the infection do not halt PTB (Gravett et al., 2007). Once the fetal membranes rupture, they are beyond rescue as there is no commonly used therapy to repair the ruptured regions, although some strategies like the Amniopatch appear promising (Deprest et al., 2011). Thus, there is a need to improve our understanding of this phenomenon, so that we can identify two groups of pPROM patients, those at risk for pPROM after infection and those at risk for non-infectious pPROM. Compounding this intricate challenge is that there are gaps in our fundamental knowledge as to how the fetal membranes weaken at the end of a normal pregnancy. Our lack of understanding of how normal membrane rupture occurs, impedes our ability to determine how this normal mechanism digresses during pPROM.

The importance of finding new therapeutic targets for the prevention of PTB, and also improving our understanding of basic parturition mechanisms, including rupture of the fetal membranes cannot be overstated. This is because much of the impact of PTB in the United States is borne by our minority populations. Americans who are members of racial and ethnic minority groups, (African Americans, American Indians and Alaska Natives, Asian Americans, Hispanics or Latinos, Native Hawaiians, and other Pacific Islanders), are more likely than Caucasians to have poor health and to die prematurely (CDC, 2020)^1^. States that have the highest rates of PTB disparity typically have large minority populations. Indeed, data from the March of Dimes mirrors this, showing that Hawai’i was ranked the 50th state in terms of PTB as a health disparity (March of Dimes Perstats^2^). The infant mortality rate is twice as high for Native Hawaiian mothers compared to whites and 43.9% of the cause of this infant mortality is PTB related (Hirai et al., 2013). Contributing to the lack of progress in Hawai’i is the lack of ethnic disaggregation, masking valuable information (Park et al., 2009; Tsark and Braun, 2009) as many established health disparities, including PTB, differentially affect ethnic groups within this population pool (Braun et al., 1996). In addition, we have no data on specific incidence of pPROM, versus other etiologies of PTB, although it is frequently seen in the clinic. It is likely that this is due to the general lack of focus on the importance of the fetal membranes in pregnancy outcomes, that is also seen in the other states. In Hawaii, like the rest of the United States, African American mothers have the highest rates of prematurity (13.8%) (March of Dimes Perstats: see text footnote 2). However, they only constitute 2.2% of the population (United States census data^3^). In other United States states African Americans constitute a much larger percentage of the population and consistently have the highest prematurity rate (Schaaf et al., 2013). These studies are typically controlled for socioeconomic and demographic confounders and therefore to improve our understanding of the underlying cause, future studies need to focus on determining the risk factors for specific ethnic groups.

## The Onset of Fetal Membrane Weakening May Be Triggered by Cellular Stress

One of the fundamental remaining questions in the field of parturition research is how the tissues of pregnancy switch from a relatively “quiescent” state that favors the maintenance of the pregnancy, to one that is “reactive” in preparation for the delivery of the fetus. Animals other than humans and non-human primates experience a drop-in progesterone level but this does not appear to happen in the same way in humans (Menon et al., 2016a). In order to increase our understanding of the differences in the mechanism, studies have focused on areas of enquiry that may lead to “functional” progesterone withdrawal, such as the role of prostaglandin receptors (Nadeem et al., 2016; Patel et al., 2018) and the minutiae of inflammation control by cytokine cascades and specific transcription factors (Lappas et al., 2008; Paulesu et al., 2010). Understating the trigger for labor onset is important for us to decipher how this may deviate in patients with PTB. It is also important to know how this labor mechanism interfaces with the trigger for the initiation of fetal membrane remodeling and weakening, another pathway that is poorly understood.

**FIGURE 1:**
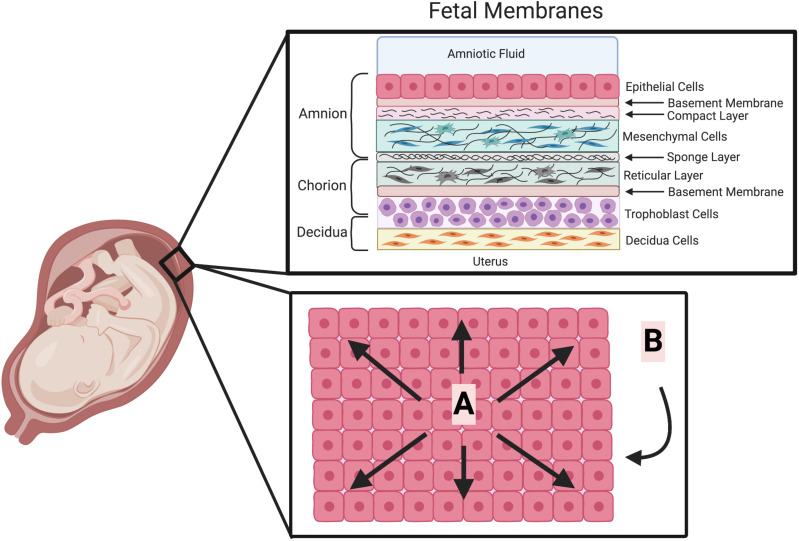
The cellular structure of the fetal membranes and their biophysical forces. The human fetal membranes consist of a multi-layered structure which surround the fetus in utero. It is composed of the amnion, chorion and decidua (upper box). Biophysical forces are placed on these fetal membranes (lower box depicted looking down onto the apical surface of the amnion epithelium). **(A)** Linear physical forces due to multi-directional stretching of the fetal membranes adherent to the decidua. **(B)** Dynamic physical forces on the fetal membranes due to fetal movements, Braxton Hicks contractions and eventually labor. (This Figure was created using BioRender).

The idea that cellular stress is the trigger for both fetal membrane weakening, and labor, has been gaining traction (Menon et al., 2016b). Suggested stressors for this mechanism have included; stretch/distension (Figure 1) of fetal membranes (Millar et al., 2000; Joyce et al., 2016) and myometrium (Waldorf et al., 2015), and also general hypoxia/oxidative stress in all of the tissues of pregnancy. Both of these stressors are known to increase in the human fetal membranes with gestational age or labor (Chai et al., 2012; Joyce et al., 2016), and also to stimulate inflammation (Kendal-Wright, 2007; Menon and Richardson, 2017). This has been demonstrated in all of the tissues of pregnancy and pregnancy complications result from the altered levels of cell stress in these tissues (Duhig et al., 2016). Indeed, several studies have shown that oxidative stress is linked to cell aging and senescence in cells of the amnion, directly leading to increased inflammation (Menon et al., 2017; Menon, 2019). It has also been shown to lead to epithelial to mesenchymal transition in the amnion, which can also play a role in the maintenance of the integrity of this tissue (Richardson et al., 2020). Other distinct types of cellular stress that have also been the subject of study in the human fetal membranes, including, Endoplasmic Reticulum Stress (Liong and Lappas, 2014) and Mitochondrial Stress (Than et al., 2009). In addition, cells can also respond to stress in a variety of way such as initiating, the heat shock response, the unfolding protein response or a DNA damage response (Fulda et al., 2010). Therefore, there are many specific pathways and mechanisms that constitute the wide umbrella term “cell stress,” these should be further investigated to elucidate their contribution to the inflammation and cellular responses seen as the fetal membranes weaken.

One of the ways in which cell stress may lead to inflammation is through the production of Danger Associated Molecular Patters (DAMPs), also known as Alarmins (Sheller-Miller et al., 2017). These molecules typically have a different specific function during normal cellular activity, but when the cell detects a stress stimulus, they are activated now functioning to signal “the alarm.”. Many different molecules are classified as DAMPs, including various heat shock proteins (HSP), extracellular matrix (ECM) breakdown products, and nucleic acid fragments (Table 1; Patel et al., 2018). DAMPs are already known to have a role in a wide range of other diseases with strong inflammatory signatures, such as, autoimmune diseases (Systemic Lupus Erythematosus, Rheumatoid Arthritis), Osteoarthritis, cardiovascular diseases, neurodegenerative diseases and cancer (Roh and Sohn, 2018). Here, they perpetuate a positive-feedback cycle of cellular damage, inflammation and then more cellular damage (Roh and Sohn, 2018). DAMPs are known to activate various Pattern Recognition Receptors (PRRs), including the Toll-like receptor (TLRs) family (Takeda et al., 2003; Kawai and Akira, 2010) and through these receptors, they can cause the activation of the pro-inflammatory transcription factor Nuclear Factor Kappa-B (NF-κB), changes in the levels of Matrix Metalloproteinases (MMP) and stimulate apoptosis (Roh and Sohn, 2018). Due to the large number of wide-ranging biomolecules acting as DAMPs (Table 1) and the large number of different receptors involved, they produce their effects by working through a complex number of distinct signaling pathways.

**TABLE 1:** Summary of key Danger Associated Molecular Patterns, their receptors and mechanisms.

Origin	DAMP	Receptor	Function (standard font = pro-inflammatory; italics = anti-inflammatory)	References
Extracellular Matrix	Aggrecan 32 mer fragment	TLR2	(iNOS), CCL2, IL-1α, IL-6, MMP12 MMP13 and ADAMTS5	Stevens et al., 2008
	Biglycan	TLR2/4	Increases levels of reactive oxygen species, CXCL-1, CCL2 and HSP70, and activates NLRP3 inflammasome by Caspase-1 and the maturation of IL-1β *Activating a TLR co-adaptor that activates IFN1 signaling*	Frey et al., 2013; Schaefer, 2014; Roedig et al., 2019
	Decorin	TLR2/4	Decreases TGFβ1 and IL-10 and increases levels of apoptosis	Merline et al., 2011
	*Fibronectin	TLR2/4	Promotes pro-inflammatory mediators and phagocytosis by macrophages	Haruta et al., 2013; Fei et al., 2018
	Fibrinogen	TLR4	Activation of monocytes	Al-Ofi et al., 2014
	LMW-HA	TLR2/4	Activates NLRP3 inflammasome by Caspase-1 and the maturation of IL-1β. Activates NF-kB	Merline et al., 2011
	HMW-HA	TLR2	*Activates a TLR co-adaptor that activates IFN1 signaling*	Scheibner et al., 2006; Frey et al., 2013
	Heparin sulfate	TLR4, RAGE	Activation of NF-kB	Xu et al., 2011
	Tenascin C	TLR4	Synthesis of pro-inflammatory cytokines	Midwood et al., 2009
	Versican	TLR2/4 CD14	IL-6, IL-1β, Il-12 and CCL2 production *Increasing IL-6 (anti-inflammatory pathways), IL-10*	Wight et al., 2014
Cytosolic	ATP	P2XR P2YR	Attracts macrophages by inflammasome activation MAPK wound healing response	Venereau et al., 2015
	Cyclophilin	CD 147	Chemotaxis and the production of pro-inflammatory factors	Burkrinsky, 2014
	F-actin	DNGR1	DNGR1 recognizes the released F-actin, which causes the uptake of damaged or dead cells	Brown, 2012
	Heat Shock Protein	TLR2/4	MyD88 dependent activation of NF-kB	Tolle and Standiford, 2013; Relja et al., 2018
	S100 proteins	TLR4, CD147 RAGE	Leads to apoptosis and activates ERK and NF-kB or AP1	Ghavami et al., 2010; Xia et al., 2018
	*Soluble amyloid beta	TLR2/4	Enhanced TNF driven inflammation	Wang et al., 2019
	Uric Acid	NLRP3	Inflammasome activation and induction of IL-1β maturation	Braga et al., 2017
Mitochondrial	mtDNA	TLR9	p38 MAPK and NF-kB activation	Zhang et al., 2010; Zhang et al., 2014; Magna and Pisetsky, 2016; Bao et al., 2016
Nuclear	*Cell free DNA	TLR9	Activation of NF-kB and AP1	Magna and Pisetsky, 2016
	Circulating Histones	TLR9	Inflammation through the activation of NF-kB	Huang et al., 2011; Kawai et al., 2016
	Extracellular self RNA	TLR7 TLR3	Sensitizes other TLR working synergistically with their other ligands MAPK, NF-κB, and IRF-5/7 pathways through MyD88 signaling	Karikó et al., 2004; Cavassani et al., 2008; Thompson et al., 2011; Noll et al., 2017; Petes et al., 2017
	*HMGB1	TLR2 TLR4 TLR9	Activation of NF-kB, and MAPK signaling though ERK and p38, release of MMPs	Qin et al., 2006; Nie et al., 2016
				Menon et al., 2011; Bredeson et al., 2014; Plazyo et al., 2016
	IL-1α	IL-1R	MAPK signaling and NF-kB activation	Betheloot and Latz, 2017
	IL-33	ST2	NF-kB activation and TNF production	Isnadi et al., 2018
	SAP130	Mincle	Triggering pro-inflammatory cytokine secretion	Zhou et al., 2016; Patkin et al., 2017

* denotes has been studied in the fetal membranes or cells of the fetal membranes. ADAMTS5, ADAM metallopeptidase with Thrombospondin type 1 motif 5; AP-1, Activator protein 1; CD, Cluster Differentiation; DNGR1, Dendritic cell natural killer lectin group receptor-1; ERK, extracellular-signal-regulated kinase; HSP, Heat Shock protein; IFN1, Interferon; IL, Interleukin; iNOS, Inducible Nitric Oxide; IRF, Interferon regulatory factors; MMP, Matrix Metalloproteinase; MyD88, myeloid differentiation primary response protein 88; NF-kB, Nuclear Factor Kappa B; NLRP3, LRR- and pyrin domain-containing protein 3; RAGE, advanced glycation end products; ST2, suppression of tumorigenicity 2; TGF, Transforming growth factor; TLR, Toll-like receptor; TNF, Tumor Necrosis Factor.

## The Weakening and Subsequent Rupture of the Human Fetal Membranes Is Dependent on Biophysical and Biochemical Change

The fetal membranes are a multilayered structure composed of various cell types and associated ECM (Figure 1). The normal rupture of these membranes is currently thought to be the result of both physical forces and biochemical changes. The physical properties of fetal membrane strength are known to originate from the layer closest to the fluid and fetus (Figure 1), the amnion (Arikat et al., 2006). The strength of this tissue is undoubtedly derived from the combination of its layers working in concert. Some of this may come from the interface between the amnion and chorion. This region in the amnion is described as a spongy layer that is ECM rich (Figure 1), consisting of proteoglycans, glycoproteins and type III collagen (Strauss, 2013). The interface between this and the chorion consists of a gelatinous substance made up of hyaluronan, decorin, buglycan and collagen that mediates the separation of the amnion and chorion prior to fetal membrane rupture (Meinert et al., 2001, 2007). This separation is the first step in the weakening of the fetal membranes (Arikat et al., 2006). The chorion adheres to the decidua between weeks 14 and 16 of pregnancy, by the degeneration of the capsular decidua and fusion of the chorion with the parietal decidua (Genbacev et al., 2015). Thus, this integration with the maternal tissues may also provide some strength to the tissue. However, the fetal membranes typically rupture in the region that is above the cervix (Malak and Bell, 1994; Strauss, 2013). *In vitro* it has been shown that after its separation from the amnion, this chorion layer is next to rupture (Arikat et al., 2006). Therefore, as the amnion layer is last to rupture in this sequence, after a notable period of deformation, it is widely accepted that the ECM rich compact layer containing amnion mesenchymal cells (AMC) (Figure 1) accounts for the strength and maintains the integrity of this tissue (Arikat et al., 2006).

An increase in apoptosis (Fortunado et al., 2000; Hsu et al., 2000; Kumagai et al., 2001) and changes in the levels of MMPs (Cockle et al., 2007) are central to the biochemical component of the changes that occur in the fetal membranes before their rupture. Although cell death in the form of apoptosis is recognized as important for the weakening process, it is thought that these cells can also die through autophagy (Shen et al., 2008; Mi et al., 2017) and perhaps necrosis (Menon and Richardson, 2017), as both of these forms of cell death are also known to be the result of cell stress (Fulda et al., 2010). In addition, it is known that necrosis can occur as the result of TLR activation in other cells (Meylan and Tschopp, 2005). Although cellular survival is obviously directly linked to the maintenance of the integrity of the amnion, its physical strength is dependent on the synthesis and degradation of the components of the ECM (El Khwad et al., 2005; Anum et al., 2009) controlled by resident cells (Parry and Strauss, 1988). Indeed, women with connective tissue disorders and related diseases are at an increased risk for complications during pregnancy, including pPROM (Anum et al., 2009). Support for this mechanism has come from several research groups as they have biochemically and mechanically identified a “zone of altered morphology” (ZAM) in the human fetal membranes (McParland et al., 2003; El Khwad et al., 2005; Osman et al., 2006; Reti et al., 2007). The ZAM constitutes a discrete zone of weakness overlying the cervix characterized by several features; an increased thickness and swelling of the connective tissue layer, a reduction in both the cytotrophoblast and decidual layers, and a reduced overall thickness of the supracervical membranes that exhibits increased ECM remodeling (Lappas et al., 2008), and apoptosis (Shen et al., 2008). It is also known that inflammation in the form of increased cytokine secretion and signaling are also involved in the initiation and progression of membrane rupture both at term and preterm. This is particularly evident when associated with intrauterine infection and chorioamnionitis (Bowen et al., 2002). However, it is important to note that inflammation in the absence of infection, in the form of what has been coined “sterile inflammation,” leads to pPROM and normal rupture of the membranes (Shim et al., 2004). Roles for several key pro-inflammatory cytokines; Interleukin 1β (IL-1β), IL-6, IL-8, and Tumor Necrosis Factor-α (TNF-α) in parturition are apparent. Their increase in abundance in gestationally advanced fetal membranes is not only associated with labor (Keelan et al., 1999) but they have also all been demonstrated to independently increase the synthesis of MMPs (Bowen et al., 2002). Additionally, many of these cytokines can cause the translocation of the pro-inflammatory transcription factor NF-κB thus leading to further increases in inflammatory mediators. This provides a pathway of pro-inflammatory self-induction (Christiaens et al., 2008) that is thought to terminate with delivery of the fetus. This can be exemplified by the chemokine IL-8, which leads to the increased infiltration of polymorphonuclear leukocytes, which can further contribute to the increase in inflammation in a feed-forward manner and can lead to birth. In further support of a central role for cytokine-induced cascades in membrane weakening, TNF-α and IL-1β have been shown to directly cause significant weakening of fetal membranes, inducing the biochemical markers characteristic of the ZAM (Kumar et al., 2006).

In addition to the polymorphonuclear leukocytes that are attracted to the tissue by chemokines, an infectious inflammatory response leads also leads to the recruitment of macrophages. These produce cytokines, MMPs, and prostaglandins, which increase the risk of pPROM (Parry and Strauss, 1988). In addition, stimulated monocytes in human chorionic cells produce the inflammatory cytokines IL-1α and TNFα, which result in the increased expression of MMP-1 and MMP-3 (Katsura et al., 1989; So et al., 1992). In fetal membranes with chorioamionitis, adhesive granulocytes have also been noted adjacent to apoptotic amnion epithelial cells (AECs) near the rupture site (Leppert et al., 1996). Together these data illustrate how immune cells promote cellular changes within the fetal membranes by driving inflammation, and breaking down ECM through the production of MMPs, predisposing the tissue for rupture. However, more recently it has been demonstrated that immune cells may also have fetal membrane healing properties through the migration of macrophages from the amniotic fluid to a rupture site in the amnion (Mogami et al., 2017). These cells were seen to induce wound healing by secreting IL-1β and TNFα and stimulating epithelial to mesenchymal transition (Mogami et al., 2017).

It is thought that the forces produced by the cell stressor distension, may be the link between the biochemical and biophysical changes seen in the fetal membranes toward term. This was originally based on the observation that human pregnancies with more than one fetus often result in premature delivery (Keith and Oleszczuk, 2002). The insertion and subsequent slow inflation of a balloon above the cervix in humans is also known to induce labor (Manabe et al., 1985). This led to the study of the distension of the uterus to discern the resultant biochemical changes and how they might lead to the activation of uterine contraction (Shynlova et al., 2009). Less work has been performed studying the effect of distension on the fetal membranes although it has been clearly shown that they are massively stretched *in vivo* at term (Millar et al., 2000; Joyce et al., 2016). It is assumed that this is the result of the combination of their adherence to the uterine wall and the termination of cellular proliferation, halting their further growth, at the beginning of the third trimester (Figure 1). Work performed stretching both the uterus, pieces of fetal membranes (Nemeth et al., 2000) or cells of the amnion (Kendal-Wright et al., 2008, 2010) show that this stimulus is able to induce pro-inflammatory cytokine production and secretion, and can also regulate apoptosis (Kendal-Wright et al., 2008; Poženel et al., 2019). Thus, the distension of the fetal membranes in normal term pregnancies and its over distension in PTB, can lead to its inflammatory signature. This distension also constitutes a significant source of cellular stress through physical strain. Interestingly, our recently collected data confirms that cellular distension of cells of the amnion *in vitro* can indeed act as a cell stressor, increasing the secretion of the DAMP, High mobility group box 1 (HMGB1) (Norman Ing et al., 2019).

## Danger Associated Molecular Patterns Are a Large Group of Biomolecules With Distinct Cellular Compartmentalization

Danger Associated Molecular Patterns are a wide-ranging group of biomolecules, originating from various cellular compartments. These molecules were classified as DAMPs when released, activated or secreted in response to tissue injury, and by damaged or dying cells (Schaefer, 2014). They can originate from nuclear or intracellular location, or cleaved from ECM. They have a wide range of effects resultant from their interaction with PRR on both immune cells and endogenous cells of organs (Table 1). The intention here is not to discuss an exhaustive list of all those that have been identified to date, but to briefly highlight the distinct origins of DAMPs and discuss what is known about them in the fetal membranes (Table 1).

The ECM has an important role in shaping the innate immune response, it is dynamic, not simply a static network that provides tissue integrity and strength. The majority of DAMPs coming from the ECM are derived from proteoglycan or glycoprotein (Table 1), and are typically released by the cleavage by MMPs, Hyaluronidase, and Heparanase (Gaudet and Popovich, 2014). However, they can also be *de novo* synthesized or released by unfolding due to mechanical stimulation (Smith et al., 2007). When released, they function to trigger sterile inflammation or prolong pathogen-induced responses by “fine-tuning” the production of inflammatory mediators (Frevert et al., 2018). Some are known to promote inflammation, whereas others are also anti-inflammatory (Frevert et al., 2018). These diverging roles are dependent on the activation of specific signaling profiles working through specific PRRs. Thus, these DAMPs work to modulate inflammation by their interaction with a range of receptors including; TLRs (TLR2 and TLR4), RIG-1-like receptors (RLRs), NOD-like receptors (NLRs), receptor for advanced glycation end products (RAGE), integrins and cluster differentiation 44 (CD44). Although their role has not been directly studied in the fetal membranes, it is reasonable, given that the amnion that provides the strength of the tissue is ECM rich (Figure 1) and is subject to cell stress in the form of distension toward term, that these molecules could have a key role in driving the weakening mechanisms of this tissue toward the end of gestation.

A wide range of DAMPs from various intracellular origins have also been characterized (Table 1). They can be cytosolic, endoplasmic, or they can also be released from granules or the plasma membrane. However, one of the most important unifying characteristics of this group of DAMPs is that when “activated” by cell stress, they typically change cellular compartment and lead to differential signaling events, compared to those seen during their normal biological role in “unstressed” conditions. In addition, various proteins and nucleic acids from the nucleus and mitochondria have been widely studied for their DAMP roles (Table 1). This group of DAMPs include but are not limited to; HMGB1, circulating histones, various interleukins, Free DNA, mitochondrial DNA, and self-extracellular RNA. The variety of DAMPs provide a range of receptor specificity and pro/anti-inflammatory functionality (Table 1).

## Danger Associated Molecular Patterns and Pathogen Associated Molecular Patterns Activate Pattern Recognition Receptors

It is clear that DAMPs cause inflammation in other tissues, therefore we believe they could be the key regulators that begin the weakening of the fetal membranes by driving inflammation during this process too. They elicit their effects through numerous receptors known as PRRs. One of the largest groups of receptors that belong to this group is the TLRs. They are a distinct class of germline-encoded PPRs that initiate the innate immune response for the initial detection of a pathogen (West et al., 2006). They are located on cell surfaces or within endosomes and generally have a conserved function to protect against pathogens via activation of downstream signaling pathways (Kawai and Akira, 2010). Several roles of TLRs have been identified including the clearance of pathogenic microbes, protection of endogenous threats, and regulation of the innate and adaptive immune response (Hug et al., 2018). There are ten TLR isoforms (TLR1–10) in humans that are expressed on immune and non-immune cells including macrophages, fibroblasts, epithelial cells, and endothelial cells (Kumar et al., 2009). TLR1, TLR2, TLR4, TLR5, and TLR6 are expressed on the cell surface, whereas TLR3, TLR7, TLR8, and TLR9 are expressed in endosomes (Takeda, 2004; Kumar et al., 2009). TLR10 is a distinct receptor, in that it is the only TLR known to act as inhibitory protein through the induction of anti-inflammatory cytokine IL-1Ra (Oosting et al., 2014).

The cell surface TLRs mainly recognize PAMPs generated from cell wall components and flagellin from gram positive and gram-negative bacteria, yeast, and fungi (Chaturvedi and Pierce, 2009). Interestingly, the recognition of DAMPs by these cell surface TLRs are shown to require different co-receptors and accessory molecules to the PAMPs. The endogenous ligands of these receptors appear to be limited, perhaps to help constrain TLR responses so that they can sufficiently function for pathogenic recognition, without causing detrimental responses to the host (Miyake, 2007). The intracellular TLRs (TLR3, 7, 8, 9) function within endolysosomal compartments and detect foreign nucleic acids that are signatures often belonging to invading viruses and microbes (Blasius and Beutler, 2010). Generally, it is thought that the endosomal TLRs are able to distinguish between host and foreign nucleic acids, although there is mounting evidence that some TLRs may not have this ability, and cannot discriminate between the nucleic acid molecules of host and microbial origin (Blasius and Beutler, 2010). It is known that endogenous mRNA and RNA from necrotic cells can also activate the intracellular TLR mediated pathway (Karikó et al., 2004; Cavassani et al., 2008; Thompson et al., 2011), resulting in an antiviral and pro-inflammatory response via Interferon (IFN) and cytokine induction (Perales-Linares and Navas-Martin, 2013).

Although the family of TLRs were originally identified for their abilities to recognize and mediate signaling pathways for a variety of microbial components (Poltorak, 1998; Takeuchi et al., 1999, 2001, 2002; Hemmi et al., 2000; Alexopoulou et al., 2001; Hayashi et al., 2001; Lund et al., 2004) many studies have shown that they are important in the detection of endogenous DAMP molecules (Table 1; Beg, 2002; Wallin et al., 2002). This is where the function of PAMPs and DAMPs differ, as DAMPs are also necessary for tissue repair. Further exploration into the exogenous (PAMP) and endogenous (DAMP) ligand activation of the TLR family may provide new and effective therapies to mediate the negative effects of TLR-driven inflammation.

**FIGURE 2:**
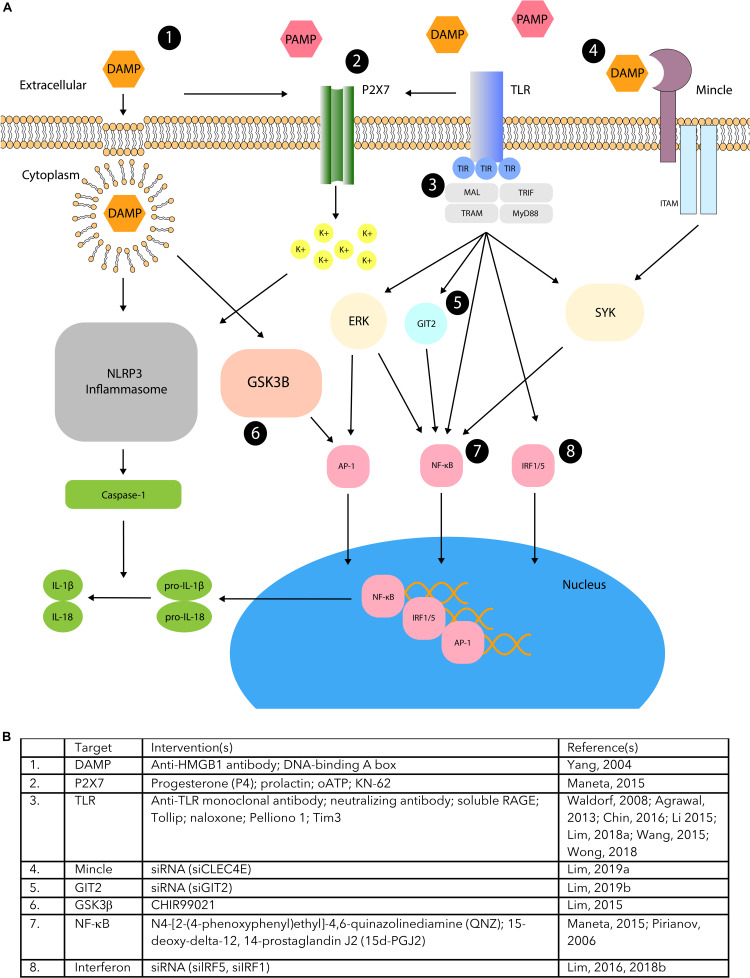
DAMP/PAMP PPR pathway manipulation. **(A)** TLR, Mincle, and P2X7 receptor activation regulated by DAMP and PAMP signaling have been shown to stimulate downstream pathways involving various modulatory proteins (ERK, GSK3B, GIT2, SYK). This may activate AP-1, NFkB, IRF1/5, or inflammasome activation that leads to induction of pro-inflammatory cytokines. Studies have demonstrated different areas of modulation (1–8) that can manipulate DAMP/PAMP mediated inflammation associated with PTB. **(B)** The supporting table lists several interventions that demonstrate the manipulation of the DAMP/PAMP mediated pathways that correspond to the different areas of modulation.

Several PRRs and co-receptors other than the family of TLR have also been shown to be important for the function of DAMPs and PAMPs (Takeuchi and Akira, 2010). These include, but are not limited to, RLRs, NLRs, RAGE, C-type lectin receptors (CLRs), P2X purinoceptor 7 receptors (P2X7Rs), LRR- and pyrin domain-containing protein 3 (NLRP3) inflammasome, and distinct members of the cluster differentiation receptor family. Similarly, to TLRs, all of these receptors are responsible for triggering distinct inflammatory cascades of the immune response. Some literature suggests that the intervention of DAMP and PAMP driven inflammation through these receptors, and their specific signaling signatures, may ablate the negative effects of associated pathologies (Figure 2).

## Danger Associated Molecular Patterns and Pattern Associated Molecular Patterns in the Fetal Membranes

There is a growing body of evidence demonstrating that DAMPs and PAMPs work via a similar set of PRRs to guide the responses of innate immune system. However, much of what we understand about the potential of DAMPs to cause fetal membrane weakening and pPROM comes from work studying the effect of PAMPs in this and other tissues. However, it cannot be assumed that the signaling events and downstream outcomes between these two groups of biomolecules (DAMPs and PAMPs), are identical, even during their activation of the same receptor. Regardless, work on the role of PAMPs in the fetal membranes has been able to provide us with insights as to how DAMPs may also function, providing foundational data that can guide our understanding of their potential as directors of fetal membrane weakening. There is a clear link between fetal membrane weakening and PAMP driven inflammation in PTB with pPROM. Indeed, TLR-mediated PTB in response to PAMPs have been studied and extensively reviewed elsewhere (Thaxton et al., 2010). Thus, the intention of this review is to discuss the work most relevant to and that was performed in the fetal membranes.

### PAMP Activation of Inflammation in the Fetal Membranes

Inflammation initiated by bacterial infection causes PTB by preterm labor and pPROM. The initiation of this inflammation is caused by different PAMPs upon their interaction with their specific TLR; for example, lipopolysaccharide (LPS) with TLR4. To determine the role of PAMP ligands specifically in fetal membranes, numerous *ex-vivo* tissue explant studies have been completed. Under normal physiological conditions the fetal membranes express all of the TLR isoforms (1-10) that could be activated and lead to specific pro-inflammatory signatures. Indeed, the treatment of fetal membrane explants with bacterial TLR agonists; peptidoglycan (TLR2), LPS (TLR4), and flagellin (TLR5) all produce a pro-inflammatory response with increased production of various cytokines, including; IL-1β, IL-6, IL-8, IL-10, granulocyte colony stimulating factor (G-CSF), macrophage inflammatory protein (MIP-1A) and Regulated upon Activation Normal T Expressed and Secreted (RANTES) (Hoang et al., 2014).

Not only do bacterial ligands lead to infection driven inflammation but they also lead to increases in the receptors that detect them. This was demonstrated in normal human fetal membranes, where the levels of TLR expression changed upon exposure to various bacteria associated with PTB pPROM. Specifically, treatment with the bacterial stimuli *Mycoplasma hominis* lead to increased expression of TLR 4, 6, and 8. Increased expression of TLR8 was also seen with *Ureaplasma parvum* and increased expression of TLR7 upon treatment with *Porphyromonas gingivalis*. Interestingly, gram negative *E. Coli* significantly decreased TLR10 expression rather than increase TLR expression as demonstrated by the other bacterial stimuli. This is of note as TLR10 is still considered an orphan receptor and therefore its function is not well understood. These data suggest that fetal membranes vary their TLR expression levels upon treatment with bacterial ligands. This observation may be used in the future to indicate the severity of infection and differences in the receptor expression level will likely further direct the magnitude of an inflammatory immune response (Abrahams et al., 2013). In further support of the bacterial ligands affecting the levels of the TLRs, fetal membranes with chorioamnionitis, have also been shown to have differential expression of TLRs. The increased expression of *TLR1* at the gene level in preterm fetal membranes with histological chorioamnionitis has been described (Waring et al., 2015), as have increases in TLR1, TLR2, TLR4, and TLR6 in chorioamniotic fetal membranes (Moco et al., 2013). Together these data suggest a role for these receptors not only in the surveillance of bacteria in this tissue but also that they adapt to the bacterial challenge.

Chorioamnionitis is not the only route to bacterial infection in the fetal membranes. Periodontal disease is a chronic inflammatory disease caused by multiple strains of bacteria, several of which have been detected in amniotic fluid, placenta and fetal membranes. It is now accepted that there is a link between these bacterial and several pregnancy complications including PTB. *P. gingivalis* has been detected in chorionic tissue and LPS derived from it, used to treat chorion derived cells. This resulted in increased expression of TLR2 causing the increased production of IL-6 and IL-8. To further study this relationship, TLR-2 gene-silenced chorionic derived cells demonstrated a reduction in IL-6 and IL-8 secretion. Taken together this suggests an important role for TLR2 in periodontic bacterial signaling in the fetal membranes (Hasegawa-Nakamura et al., 2011). In support of this, the periodontal bacteria *Fusobacterium nucleatum* has also been detected in chorionic tissue from high-risk pregnant women. Similarly, to the previous study, it was then used to treat chorionic cells where it was also able to increase inflammation by increasing IL-6 and corticotrophin-releasing hormone (CRH) secretion via TLR2 and TLR4 activation (Tateishi et al., 2012). These data indicate that periodontal bacteria act similarly to other PAMPs in these tissues as they find their way into the amniotic fluid, placenta and fetal membranes, where they activate the TLR system and downstream inflammation.

Although humans are not thought to experience the sharp drop in progesterone that is thought to initiate the onset of parturition in other animals (Menon et al., 2016a), its administration is used to maintain pregnancy and lower the risk of PTB (Chenung et al., 2020) as it is thought that humans may experience a “functional” progesterone withdrawal (Nadeem et al., 2016). Progesterone (P4) has been shown to have a protective role in fetal membranes by reducing pro-inflammatory cytokine production upon LPS treatment (Flores-Espinosa et al., 2014). It does this in part by significantly reducing TLR4 expression upon LPS treatment. This directly results in the reduced secretion of the pro-inflammatory cytokines TNFα, IL-6, and β-defensin 2 (HBD2) (Flores-Espinosa et al., 2014). Therefore, this intriguing mechanism could be a way for progesterone to decrease the ability of this tissue to detect DAMPs until its functional levels decrease toward the end of gestation.

Animal models have also been used to determine how PAMPs induce inflammation leading to different pregnancy complications. They have also provided models of PTB that can be used to test novel therapeutic targets. In humans, the histological assessment of preterm fetal membranes detected TLR4 expression in the fundus and low segment suggesting a role in PTB (Choi et al., 2012). This receptor’s role was further investigated in an infection, LPS-induced PTB mouse model. The TLR4 antagonist (+)-naloxone, was able to suppress the expression the inflammatory cytokines IL-1β, IL-6, TNFα, and IL-10 and prevent PTB. Another study also established the role for TLR4 in PTB by using a mouse model by inducing infection with an LPS variant, heat killed *E. Coli* (HKE). HKE treatment induced PTB in all TLR4 normal mice compared to none of the TLR4 mutant mice. In addition, another study induced PTB in CD1 mice by administering intrauterine injections of saline, peptidoglycan (PGN, TLR2 agonist) or polyinosinic-polycytidylic acid (Poly I:C, TLR3 agonist) (Jaiswal et al., 2013). The regulation of a2 isoform of V-ATPase (a2V) has a role in pregnancy and was assessed post exposure to these two ligands, PGN and Poly I:C. This led to significantly decreased expression of a2V in the fetal membranes and play a role in the induction of PTB in mice (Jaiswal et al., 2013).

Collectively, several mouse studies have provided evidence that demonstrate the importance of bacterial PAMP/TLR driven inflammation to cause PTB. Providing model systems that can be continued to be used to study future therapies to address PTB by these pathways. Indeed, models activating TLRs by ligand injection (Kaga et al., 1996; Wang and Hirsch, 2003) or placental stimulation (Liu et al., 2007; Koga et al., 2009) are also models for PTB.

### PAMP Stimulation of Amnion Epithelial Cells Isolated From Fetal Membranes

Human AECs form the most superficial layer of the fetal membranes functioning as an immunological barrier for the fetus against intra-amniotic infection. Their response to pregnancy specific PAMP ligands has been studied to help us understand the contribution of these cells to inflammation in the fetal membranes but also to their production of pro-inflammatory signaling molecules that may also communicate with the growing fetus. Under normal physiological conditions, these cells express TLR 1-10 and can be activated by the PAMPs that reach them. TLR5 and TLR 2/6 activation by PAMP the ligands, Flagellin and macrophage-activating lipopeptide-2 (MALP-2), respectively, significantly increase the secretion of IL-6 and IL-8 (Gillaux et al., 2011). They also cause the nuclear translocation of NF-κB subunit p65, thus inducing a pro-inflammatory response. The activation of TLR4 by LPS, however, has also been demonstrated to induce apoptosis and decrease cell viability of these cells (Gillaux et al., 2011). However, the activation of this receptor also increases Transforming Growth factor beta 1 (TGFβ1) and prostaglandin E2 (PGE2), TNFα and IL-1β production (Motedayyen et al., 2019), which has been confirmed for TGFβ1 and PGE2 but not for IL-1β, IL-6, and IL-10 (Taheri et al., 2018). Taken together these data suggest that not only does the activation of this PAMP TLR mechanism mediate an immunological response to PAMPs in AEC, but it could contribute to the inflammation seen in the fetal membranes with PTB and may also signal to the fetus when an infection was detected. It is possible that AMC respond similarly to AEC to PAMP stimulation, however, there has been a lack of focus on this system in AMC. In addition, differences in the fundamental functions of these cells in the fetal membranes make it difficult to evaluate similarities between them. Thus, it is challenging to know how similar the pathway signaling is in this cell type, that functions to maintain the integrity of ECM in the fetal membranes.

### PAMP Stimulation of Amnion Mesenchymal Cells Isolated From Fetal Membranes

Human AMCs are located within the ECM rich layer of the amnion that is closest to the chorion in the of fetal membranes (Figure 1). This layer is responsible for the maintenance of the strength and integrity of the whole tissue. Our work has shown that the human AMCs isolated from human fetal membranes express all ten TLR isoforms (Sato et al., 2016). This suggests that similarly to AECs, AMC are able to detect PAMPs and produce a pro-inflammatory signature. However, as their location and function are different to that of AEC, the downstream consequences may also be different. We have also been able to show that indeed the TLR2/6 ligand MALP-2, could increase the pro-inflammatory cytokines and NF-κB translocation, it did not increase levels of apoptosis.

### Viral PAMP Activation of the Fetal Membranes

In addition to bacterially derived PAMPs, viral infection also elicits an immune response through the activation of TLRs. This is typically through the release and subsequent detection of viral nucleic acids. TLR 3, 7, 8, and 9 are all nucleic acid ligand response receptors that are activated upon exposure to viral nucleic acid ligands. Thus, fetal membrane explants treated with viral double stranded RNA (dsRNA) and viral single stranded RNA (ssRNA) activate TLR 3 and TLR8, respectively, and induce downstream pro-inflammatory cytokine production that result in two different distinct antiviral response profiles (Bakaysa et al., 2014). Those explants treated with Poly (I:C) (TLR3), or the viral ligands, imiquimod (TLR7), and ssRNA40 (TLR8) also caused the production of pro-inflammatory cytokines IL-6 and IL-8 (Bryant et al., 2017). Although there is little data on the response of the fetal membranes to viral PAMPs, these data support the idea that all of the viral, as well as bacterial TLR receptors are able to be activated on cells of the fetal membranes and that they could result in inflammation, potentially leading to fetal membrane weakening and rupture.

### DAMPS and Fetal Membranes

Work studying the direct effect of DAMPs or DAMP production by the fetal membranes is limited. Indeed, so far to our knowledge only four have been directly tested in the fetal membranes (Table 1). However, others that have also been shown to play a role in pregnancy are, HMGB1, cell-free fetal DNA (cffDNA), uric acid and IL-1. Little is known about the specific roles of any of these molecules in fetal membranes, and their potential as novel therapeutic targets and biomarkers in the pathologies of pregnancy.

High mobility group box 1 is the most comprehensively studied DAMP in the fetal membranes and has been shown to be a critical regulator throughout different stages of pregnancy. It activates TLRs to initiate an inflammatory immune response in a sterile environment, but it is also released by PAMP stimulation of TLRs. This was confirmed by its presence in the Amniotic fluid from preterm mothers with damaged fetal membranes due to intra-amniotic infection (Baumbusch et al., 2016). Its increase in levels also correlate with increases in IL-6 and with infection in amniotic fluid but its does not seem to increase with gestational age in this compartment. HMGB1 levels are also elevated in chorioamniotic membrane extracts from preterm labor compared to term labor (Plazyo et al., 2016) and it has been seen that this increase contributes to increased expression of pro-inflammatory cytokines (Plazyo et al., 2016). It also increases *MMP-9* gene expression and active-MMP-9 levels in chorioamniotic fetal membrane explants, compared to control treated fetal membranes (Plazyo et al., 2016). These data suggest that HMGB1 induction of MMP-9 could directly promote fetal membrane weakening. Pregnant mice treated with intra-amniotic injections of HMGB1 resulted in increased pup mortality, PTL and PTB compared to pregnant mice treated with PBS (Gomez-Lopez et al., 2016). This study did not asses the role of HMGB1 in the fetal membranes, but these data suggest HMGB1 has an important role in promoting PTB. Fetal membranes treated with exogenous HMGB1 increase TLR2 and TLR4 expression and activation of the inflammatory immune response regulator, p38 Mitogen Activated Protein Kinases (MAPK) which also leads to a senescent phenotype consistent with a sterile inflammatory response (Bredeson et al., 2014). Moreover, term fetal membranes have a phenotype consistent with senescent cells, reduced telomeres, increased activation of p38 MAPK and increased in senescence-associated beta (SA-B) galactosidase and senescence-associated secretory phenotype (SASP) gene expression found a senescent phenotype (Menon et al., 2016b). HMGB1, oxidative stress and apoptosis is also increased in normal fetal membrane explants exposed to cigarette smoke extract compared to control treated fetal membrane explants (Menon et al., 2011). In a study that also linked HMGB1 with fetal membrane weakening, fetal membranes from women who had preterm labor or pPROM were found to have increased levels of HMGB1 in their serum (Qiu et al., 2017). This suggests that HMGB1 could also be a potential biomarker for PTB. Immunofluorescent labeling of fetal membranes delivered preterm with chorioamnionitis also showed HMGB1 localized AECs, with more diffuse labeling in the myofibroblast and macrophages compared to term fetal membranes delivered at term (Romero et al., 2011). Interestingly, another study has demonstrated that microRNA 548 (miRNA) regulates the expression of HMGB1 in fetal membrane tissues with very minimal expression in preterm chorioamniotic fetal membranes (Son et al., 2019). Induced expression of miRNA 548 in isolated epithelial cells from fetal membranes decreased HMGB1 levels and pro-inflammatory cytokine levels. Together these data strongly suggest that although HMGB1 secretion is increased during the cellular stress of infection it may also have a role in a sterile immune response working in autocrine and paracrine ways that could contribute to PTB.

A possible role for serum amyloid A1 (SAA1) in ECM remodeling was assessed in amnion fetal membrane explants. Human amnion fetal membrane explants treated with SAA1 showed increased protein expression of MMP-1, MMP-8, and MMP-13 compared to control treatment (Wang et al., 2019). The increased expression of MMPs suggest a potential role for SAA1 in ECM remodeling in fetal membranes after rupture.

TLR9 recognizes unmethylated CpG-containing DNA sequences in addition to bacterial and viral DNA to activate an immune response, similarly to all the other DAMPs. cffDNA is one of the few nucleic acid DAMPs that have been studied in the context of a placental source of cffDNA. It is thought that cffDNA released from the placenta can circulate in the maternal plasma to activate TLR9 on leukocytes and macrophages. This then can induce a pro-inflammatory immune response that triggers labor, which ultimately leads to birth (Phillippe, 2014). A study in support of the importance of this group of DAMPs, demonstrated that TLR9 is expressed on fetal membranes from term placentas in the presence and absence of labor (Beck et al., 2019). Indeed, its levels are highly expressed in AEC (Gillaux et al., 2011; Sato et al., 2016) and AMC (Sato et al., 2016). Exosomes from senescent AECs are known to package cffDNA and HMGB1 that could also to signal within the fetal membranes acting as a trigger for parturition (Sheller-Miller et al., 2017). In further support of this, human peripheral blood mononuclear cells (PBMCs) treated with fetal DNA or CpG DNA increased IL-6 secretion and pregnant Bagg Albino (BALB/c) mice injected with fetal DNA, CpG DNA or LPS increased the number of resorbed fetuses. In addition, TLR9 knockout mice treated with fetal DNA showed decreased numbers of resorbed mice, whereas those treated with the TLR9 inhibitor Chloroquine reduced fetal resorptions and IL-6 production. This suggests a role for TLR9 in promoting PTB upon cffDNA treatment (Scharfe-Nugent et al., 2012). More studies are needed to show the mechanism by which cffDNA could contribute to the activation of parturition and also what role it has in the fetal membranes where the expression of its detection receptors is high in AEC and AMC (Sato et al., 2016).

To our knowledge only one study has specifically tested the activation of AMC by DAMPs. In this study the authors elegantly show that the TLR4 receptor is present and able to be activated by the DAMP, fetal fibronectin, on AMC (Haruta et al., 2013) resulting in the increase in the activity of MMP-1 and MMP-9.

It is clear that due to the high incidence of infection driven PTB, including those with pPROM, the role of TLR and their ligands in the fetal membranes with and without chorioamnionitis has been the subject of some study. To date, only a few papers have produced data linking DAMPs (as opposed to PAMPs) and the fetal membranes. These have mostly focused on the production of HMGB1, which has been confirmed to increase in amniotic fluid of human fetal membranes with pPROM (Romero et al., 2011), and is induced by intrauterine infection with LPS, in sheep (Regan et al., 2016). Even though much of the current work that is focused on DAMPs in the fetal membranes is AEC centric, the data provides strong evidence that DAMP production by AMC would produce inflammation, perhaps in an autocrine fashion, and this would likely influence the integrity of the amnion. In addition, DAMPs are produced by many of the other cells important for the maintenance of pregnancy (Phillippe, 2014; Nadeau-Vallee et al., 2016). They could also activate AMC in a paracrine fashion and contribute to the increase in inflammation and MMPs, which could ultimately lead to membrane rupture.

## DAMP/PAMP PRR Pathway Manipulation: Potential Therapeutic Strategies for Fetal Membrane Maintenance

There are a multitude of developing therapies based on PRRs recognition of their ligands for the resolution of diseases with an inflammatory signature. Indeed, in other disease and disorder fields like sepsis for example, DAMP manipulation is being tested by the antagonism of HMGB1 (Yang et al., 2005). Although this body of work is expansive, it will not be focused on here as it would warrant its own focused review article. Here we aim to describe the studies that have been specifically focused on the manipulation of this pathway in the tissues of pregnancy, including the fetal membranes where possible, and therefore show promise for future development of strategies to address the maintenance of the integrity of this tissue (Figure 2).

Signaling events activated by the DAMP and PAMP ligands are numerous, and complex, with an ever-expanding repertoire of players. Some of these molecules overlap between pathways, while others are more specific. This broad network provides numerous potential targets for manipulation and his has been explored due to their potential for other causes of PTB, but rarely for their direct effects on the fetal membranes. This is because it is highly likely that DAMPs and PAMPs working through their PRRs also have a role in myometrial activation and cervical ripening. This body of work has provided some exciting data (Figure 2) that may be used to help us discern the ability for some of the already tested strategies, or develop novel ones, that could be used to help manipulate the timing of membrane rupture too.

It has been suggested that anti-TLR monoclonal antibodies could be good therapeutic target to decrease inflammation driven adverse perinatal outcomes (Wong et al., 2018) and thus it should be considered whether they may also serve as good PTB therapies. Indeed, neutralizing antibodies against TLR4 reduce the percentages of decidual invariant natural killer cells, decreasing inflammation (Li et al., 2015). Soluble TLRs have also been suggested as potential markers or treatment sites for those conditions where they are understood to have a key role (Abrahams et al., 2013). However, it still remains to see their effects elsewhere in the other tissues when pregnant. An alternative strategy in the same vein has been to use soluble RAGE as a therapeutic tool to decrease inflammation. Pretreatment with these inhibited LPS-induced preterm uterine contractility, cytokines, and prostaglandins in Rhesus monkeys (Waldorf et al., 2008), supporting the premise that this approach has merit for further exploration in pregnancy.

An alternate approach designed to target pathways more specifically has been to knockdown downstream signaling for the PRRs. Interferon regulatory factor (IRF) 5 knockdown leads to less inflammation in myometrium (Lim et al., 2018) and thus inhibitors for IRF1 have also been suggested as a therapeutic strategy due to their direct role in TLR signaling (Lim et al., 2016). Other studies testing the potential of TLR4 inhibitors have been successful at decreasing levels of inflammation associated with its activation. Indeed, levels of Tollip, an inhibitor of TLR4 that is known to correlate with PE severity (Nizyaeva et al., 2019) and the TLR4 antagonist (+)-naloxone (Chin et al., 2016) were shown to suppress cytokine expression. Although these studies were performed in the placenta and myometrium/decidua, respectively, they provide promising data for developing this approach in fetal membranes.

Other work has focused specifically on PAMP stimulated TLR response manipulation. Thus, several different biomolecules have been shown to inhibit inflammation or PTB in animal models due to their ability to decrease TLR ligand induced responses. Surfactant-A intrauterine injection was found to significantly decrease TLR ligand induced inflammation and inhibit preterm delivery via TLR2 (Agrawal et al., 2014). As GSK3 α and β activity is increased in fetal membranes after labor, the GSK3β inhibitor CHIR99021 was tested and found to decrease LPS stimulated pro-inflammatory cytokines, TNFα, IL-1β, IL-6, IL-8, prostaglandins and MMP-9 in myometrial cells and fetal membranes cells (Lim and Lappas, 2015). In addition, the protein Pellino 1 was shown to regulate TLR and TNF signaling, by decreasing TLR2/6, TLR3, TLR5 and the pro-inflammatory cytokines IL-8, IL-1β, and IL-6 (Lim et al., 2018). When the addition of Tim3 was tested, it was found to protect decidual cells from TLR extracellular signal-regulated protein kinase 1 and 2 (ERK1/2) dependent apoptosis and inflammation (Wang et al., 2015).

Decreases in TLR signaling by the removal of their key partners or decreasing receptor numbers have also been studied. The elimination of Mincle, a sensor for lipids, was shown to lead to decreased effect of TLR ligands to cause inflammation in uterus (Lim and Lappas, 2019). In addition, GIT2 knockdown, in myometrial and amnion cells also lead to decreased inflammation in response to TLR ligands (Lim and Lappas, 2019). The P2X7 receptor, which has been shown to regulate IL-1β release from gestational tissues working in concert with TLRs has also been studied. The inflammation it produced on stimulation with a specific receptor agonist, was inhibited by the progesterone, prolactin and an NF-κB inhibitor (Maneta et al., 2015). Finally, treatment with 15-deoxy-delta-12, 14-prostaglandin J2 (15d-PGJ2) affected TLR4 by blocking its NF-κB induced inflammation, reducing preterm labor in a mouse model (Pirianov et al., 2009).

It remains to be seen how the further development of these potential therapeutic targets will continue. With such a large number of signaling pathways, receptors and ligands involved, there are many other targets that are yet to be investigated. However, which of the manipulated mechanisms will have too many unwanted effects, or that have an unwanted global biological effect on other organs and the fetus, remains to be determined. Regardless, interest in these pathways is gathering momentum due to their potential to increase our understanding of the mechanisms at play and to prevent the rupture of the membranes. Due to their central role in infection and non-infection driven inflammation, they also have enormous potential for treatments for the other etiologies of PTB (Ekman-Ordeberg and Dubicke, 2012).

## Summary

The trigger that initiates tissue remodeling in preparation for parturition remains elusive. This missing stimulus includes that which activates the weakening cascade of the fetal membranes in preparation for its rupture. Understanding this is not only important to determine what happens in normal pregnancy but also because the timing of this event is crucial for healthy pregnancy outcomes. If rupture does not occur in a timely fashion it can result in pROM or pPROM leaving the fetus vulnerable to infection and distress. It can also precipitate PTB, as pPROM is evident in approximately one third of all cases of premature delivery. It is currently thought that the change that may switch on the process of membrane weakening is an increase in cellular stress. This is already known to increase toward the end of gestation in the form of physical distension of the fetal membrane tissue and also the build-up of ROS. It is well established that DAMPs are generated by many forms of cell stress and their general biological function is to raise the alarm within tissues. They do this by producing a number of pro-inflammatory cytokines, MMPs and often lead to apoptosis. They accomplish this through their interaction with numerous PRRs and their downstream signaling pathways, often achieving their influence on inflammation by activating the transcription factor NF-κB. Much of what we understand about the role of DAMPs, especially in the fetal membranes, comes from work in other tissue types or from consideration of data from PAMP ligand action. This is because these ligands also work through many of the same PRRs. However, these data should be viewed with caution; PAMPs and DAMPs have been seen to result in differential signaling through the same PRR. Despite this, numerous similarities that have been described in their actions. PAMPs are well established to cause infection driven membrane rupture, and so it is tempting to postulate that the DAMP counterparts are also able to do this. These molecules could therefore be the link between the biophysical and biochemical changes that happen toward term to weaken the fetal membranes. This idea has been least studied from the perspective of understanding the ECM DAMPs. Perhaps this is because they are one of the more difficult molecules to study in this tissue, given that most researchers are working on it at in term state when much of the ECM has already started to breakdown. Thus, we need to build new models to study the interaction and generation of ECM DAMPs with the AMC within this layer of the amnion. There have been several studies that have focused on understanding the potential of the manipulation of the DAMP, PAMP, PRR system, with an eye to future therapies for various PTB etiologies. Although still in their infancy, it is tempting to speculate that more work focused in this vein will indeed not only solidify the key role of these molecules in parturition, but provide much needed prevention and identification biomolecular targets to help us address the high rates of PTB in the United States.

## Author Contributions

JP contributed to all the written sections focused on the different receptors and also drew and annotated Figure 1. CS contributed to the PAMPs and DAMPs written sections and assisted with editing. CK-W contributed to all sections, tables, and figures.

## Conflict of Interest

The authors declare that the research was conducted in the absence of any commercial or financial relationships that could be construed as a potential conflict of interest.

## Footnotes

1 http://www.cdc.gov/omh/AMH/dbrf.htm

2 http://www.marchofdimes.com/peristats/Peristats.aspx (accessed May 7, 2020).

3 https://www.census.gov/quickfacts/fact/table/HI/PST045219# (accessed May 7, 2020).

## Abbreviations

15d-PGJ2, 15-deoxy-delta-12: 14-prostaglandin J2
a2V: V-ATPase
ADAMTS5: ADAM metallopeptidase with Thrombospondin type 1 motif 5
AEC: amnion epithelial cell
AMC: amnion mesenchymal cell
AP1: Activator protein 1
ATP: Adenosine tri-phosphate
CD: cluster differentiation
cffDNA: cell free fetal DNA
CLR: C-type lectin receptors
DAMP: Danger associated molecular pattern
DNGR1: Dendritic cell natural killer lectin group receptor-1
dsRNA: double stranded ribonucleic acid
ECM: Extracellular Matrix
ERK: extracellular-signal-regulated kinase
G-CSF: granulocyte colony stimulating factor
GIT2: ARF GTPase-activating protein
GSK: Glycogen synthase kinase 3
HBD2: b-defensin 2 HBD2
HKE: Heat killed *E. Coli*
HMGB1: high mobility group box 1
HMW: high molecular weight
HSP: Heat Shock protein
IFN: interferon
IL: Interleukin
iNOS: inducible nitric oxide synthase
IRF: IFN regulatory factors
IRF: interferon-regulatory factor
LMW: low molecular weight
LPS: Lipopolysaccharide
LRR: Leucine-rich repeats
MAL/TIRAP: MyD88 adaptor-like protein
MALP-2/FLS-1: diacyl lipopeptides
MAPK: mitogen activated protein kinase
MIP-1A: macrophage inflammatory protein
miRNA: micro ribonucleic acid
MMP: Matrix Metalloproteinase
mtDNA: mitochondrial deoxyribonucleic acid
MYD88: myeloid differentiation primary response protein 88
NF-kB: Nuclear Factor Kappa B
NLR: NOD-like receptors
NLRP3: LRR- and pyrin domain-containing protein 3
P2YR: P2 receptor
P2X7R: P2X purinoceptor 7 receptors
PAMP: Pattern associated molecular pattern
PBMC: peripheral blood mononuclear cell
PBS: phosphate buffered saline
PGE2: prostaglandin E2
PGN: peptidoglycan
Poly I:C: polyinosinic-polycytidylic
pPROM: preterm premature rupture of the membranes
PRRs: Pattern recognition receptors
PTB: Preterm birth
PTL: Preterm labor
RAGE: advanced glycation end products
RANTES, regulated upon activation: normal T expressed and secreted
RD: repressor domain
RLR: RIG-1-like receptors
ROS: reactive oxygen species
SAA1: serum amyloid A1
SAP130: sin3A associated protein 3A
SASP: senescence-associated secretory phenotype
ssRNA: single stranded ribonucleic acid
ST2: suppression of tumorigenicity 2
TIM: T-cell immunoglobulin and mucin-containing domain-3
TIR: Toll/IL1 receptor
TLR: Toll-like receptors
TNF: tumor necrosis factor
TRAM: TIR domain-containing adapter molecule 2
TRIF: TIR domain-containing adaptor-inducing IFN β TRIF
ZAM: zone of altered morphology.

## References

[B1] Abrahams V. M. Potter J. A. Bhat G. Peltier M. R. Saade G. Menon R. (2013). Bacterial modulation of human fetal membrane Toll-like receptor expression. *Am. J. Reprod. Immunol.* 69 33–40. 10.1111/aji.12016 22967004 PMC3535068

[B2] Agrawal V. Jaiswal M. K. Ilievski V. Beaman K. D. Jilling T. Hirsch E. (2014). Platelet-activating factor: a role in preterm delivery and an essential interaction with Toll-like receptor signaling in mice. *Biol. Reprod.* 91:119. 10.1095/biolreprod.113.116012 25253732 PMC4434927

[B3] Alexopoulou L. Holt A. C. Medzhitov R. Flavell R. A. (2001). Recognition of double-stranded RNA and activation of NF-κB by Toll-like receptor 3. *Nature* 413 732–738. 10.1038/35099560 11607032

[B4] Al-Ofi E. Coffelt S. B. Anumba D. O. (2014). Fibrinogen, an endogenous ligand of toll-like receptor 4, activates monocytes in pre-eclamptic patients. *J. Reprod. Immunol.* 103 23–28. 10.1016/j.jri.2014.02.004 24661950

[B5] Anum E. Hill L. Pandya A. Straus J. (2009). Connective tissue and related disorders and preterm birth: clues to genes contributing to prematurity. *Placenta* 30 207–215. 10.1016/j.placenta.2008.12.007 19152976 PMC2673455

[B6] Arikat S. Novince R. W. Mercer B. M. Kumar D. Fox J. M. Mansour J. M. (2006). Separation of amnion from choriodecidua is an integral event to the rupture of normal term fetal membranes and constitutes a significant component of the work required. *Am. J. Obstet. Gynecol*. 194 211–217. 10.1016/j.ajog.1005.06.083 16389034

[B7] Astern J. M. Collier A. C. Kendal-Wright C. E. (2012). Pre-B cell colony enhancing factor (PBEF/NAMPT/Visfatin) and vascular endothelial growth factor (VEGF) cooperate to increase the permeability of the human placental amnion. *Placenta* 34 42–49. 10.1016/j.placenta.2012.10.008 23151382 PMC3541826

[B8] Bakaysa S. L. Potter J. A. Hoang M. Han C. S. Guller S. Norwitz E. R. (2014). Single- and double-stranded viral RNA generate distinct cytokine and antiviral responses in human fetal membranes. *Mol. Hum. Reprod.* 20 701–708. 10.1093/molehr/gau028 24723465 PMC4072183

[B9] Bao W. Xia H. Liang Y. Ye Y. Lu Y. Xu X. (2016). Toll-like receptor 9 can be activated by endogenous mitochondrial dna to induce podocyte apoptosis. *Sci. Rep*. 6:22579. 10.1038/srep22579 26934958 PMC4776276

[B10] Baumbusch M. A. Buhimschi C. S. Oliver E. A. Zhao G. Thung S. Rood K. (2016). High mobility group box 1 (HMGB1) levels are increased in amniotic fluid of women with intra-amniotic inflammation-determined preterm birth, and the source may be the damaged fetal membranes. *Cytokine* 81 82–87. 10.1016/j.cyto.2016.02.013 26954343 PMC4803598

[B11] Beck S. Buhimschi I. A. Summerfield T. L. Ackerman W. E. Guzeloglu-Kayisli O. Kayisli U. A. (2019). Toll-like receptor 9, maternal cell-free DNA and myometrial cell response to CpG oligodeoxynucleotide stimulation. *Am. J. Rerod. Immunol.* 81 1–12. 10.1111/aji.13100 30758898 PMC6453711

[B12] Beg A. (2002). Endogenous ligands of Toll-like receptors: implications for regulating inflammatory and immune responses. *Trends Immunol.* 23 509–512. 10.1016/s1471-4906(02)02317-7 12401394

[B13] Betheloot D. Latz E. (2017). HMGB1, IL-1alpha, IL-33 and S100 proteins: dual-function alarmins. *Cell. Mol. Immunol.* 14 43–64. 10.1038/cmi.2016.34 27569562 PMC5214941

[B14] Blasius A. L. Beutler B. (2010). Intracellular toll-like receptors. *Immunity* 32 305–315. 10.1016/j.immuni.2010.03.012 20346772

[B15] Bowen J. M. Chamley L. Keelan J. A. Mitchell M. D. (2002). Cytokines of the placenta and extra-placental membranes: roles and regulation during human pregnancy and parturition. *Placenta* 23 257–273. 10.1053/plac.2001.0782 11969336

[B16] Braga T. T. Forni M. Correa-Costa M. Ramos R. N. Barbuto J. A. Branco P. (2017). Soluble uric acid activates the NLRP inflammasome. *Sci. Rep.* 7:39884. 10.1038/srep39884 28084303 PMC5233987

[B17] Braun K. L. Look M. A. Yang H. Onaka A. T. Horiuchi B. Y. (1996). Native Hawaiian mortality, 1980 and 1990. *Am. J. Public Health* 86 888–889. 8659673 10.2105/ajph.86.6.888 PMC1380415

[B18] Bredeson S. Papaconstantinou J. Deford J. H. Kechichian T. Syed T. A. Saade G. R. (2014). HMGB1 promotes a p38MAPK associated non-infectious inflammatory response pathway in human fetal membranes. *PLoS One* 9:e113799. 10.1371/journal.pone.0113799 25469638 PMC4254744

[B19] Brown G. D. (2012). Immunology: actin’ dangerously. *Nature* 485 589–590. 10.1038/485589a 22660316

[B20] Bryant A. H. Menzies G. E. Scott L. M. Spencer-Harty S. Davies L. B. Smith R. A. (2017). Human gestation-associated tissues express functional cytosolic nucleic acid sensing pattern recognition receptors. *Clin. Exp. Immunol.* 189 36–46. 10.1111/cei.12960 28295207 PMC5461091

[B21] Burkrinsky M. (2014). Extracellular cyclophilins in health and disease. *Biochim. Biophys. Acta* 1850 2087–2095. 10.1016/j.bbagen.2014.11.013 25445705 PMC4436085

[B22] Cavassani K. A. Ishii M. Wen H. Schaller M. A. Lincoln P. M. Lukacs N. W. (2008). TLR3 is an endogenous sensor of tissue necrosis during acute inflammatory events. *J. Exp. Med.* 205 2609–2621. 10.1084/jem.20081370 18838547 PMC2571935

[B23] CDC (2020). *Centers for Disease Control and Prevention.* Available online at: http://www.cdc.gov/omh/AMH/dbrf.htm (accessed May 7, 2020).

[B24] Chai M. Barker G. Menon R. Lappas M. (2012). Increased oxidative stress in human fetal membranes overlying the cervix from term non-labouring and post labour deliveries. *Placenta* 33 604–610. 10.1016/j.placenta.2012.04.014 22595042

[B25] Chaturvedi A. Pierce S. K. (2009). How location governs toll-like receptor signaling. *Traffic* 10 621–628. 10.1111/j.1600-0854.2009.00899.x 19302269 PMC2741634

[B26] Chenung K. W. Seto M. T. Y. Ng E. H. Y. (2020). Early universal use of oral progesterone for prevention of preterm births in singleton pregnancy (SINPRO study): protocol of a multicenter, randomized, double-blind, placebo-controlled trial. *Trials* 21:121. 10.1186/s13063-020-4067-z 32000820 PMC6993330

[B27] Chin P. Y. Dorian C. L. Hutchinson M. R. Olson D. M. Rice K. C. Moldenhauer L. M. (2016). Novel toll-like receptor-4 antagonist (+)-naloxone protects mice from inflammation-induced preterm birth. *Sci. Rep.* 6:36112. 10.1038/srep36112 27819333 PMC5098167

[B28] Choi S. J. Jung S. H. Eom M. Han K. H. Chung I. B. Kim S. K. (2012). Immunohistochemical distribution of toll-like receptor 4 in preterm human fetal membrane. *J. Obstet. Gynaecol. Res.* 38 108–112. 10.1111/j.1447-0756.2011.01626.x 21827576

[B29] Christiaens I. Zaragoza D. Guilbert L. Roberston S. A. Micthell B. F. Olson D. M. (2008). Inflammatory processes in preterm and term parturition. *J. Reprod. Immunol.* 79 50–57. 10.1016/j.jri.2008.04.002 18550178

[B30] Cockle J. Gopichandran N. Walker J. Levene M. I. Orsi N. M. (2007). Matrix metalloproteinases and their tissue inhibitors in preterm perinatal complications. *Reprod. Sci.* 14 629–645. 10.1177/1933719107304563 18000225

[B31] Deprest J. Emonds M. P. Richter J. DeKoninck P. Van Mieghem T. Van Schoubroeck D. (2011). Amniopatch for iatrogenic rupture of the fetal membranes. *Prenat. Diagn.* 31 661–666. 10.1002/pd.2780 21656529

[B32] Duhig K. Chappell L. C. Shennan A. H. (2016). Oxidative stress in pregnancy and reproduction. *Obstet. Med.* 9 113–116. 10.1177/1753495X16648495 27630746 PMC5010123

[B33] Ekman-Ordeberg G. Dubicke A. (2012). Preterm Cervical Ripening in humans. *Facts Views Vis. Obgyn.* 4 245–253. 24753916 PMC3987477

[B34] El Khwad M. Stetzer B. Moore R. Kumar D. Mercer B. Arikat S. (2005). Term human fetal membranes have a weak zone overlying the lower uterine pole and cervix before onset of labor. *Biol. Reprod.* 72 720–726. 10.1095/biolreprod.104.033647 15548732

[B35] Fei D. Meng X. Yu W. Yang S. Song N. CaO Y. (2018). Fibronectin (FN) cooperated with TLR2/TLR4 receptor to promote innate immune responses of macrophages via binding to integrin β1. *Virulence* 9 1588–1600. 10.1080/21505594.2018.1528841 30272511 PMC7000207

[B36] Flores-Espinosa P. Pineda-Torres M. Vega-Sanchez R. Estrada-Gutierrez G. Espejel-Nunez A. Flores-Pliego A. (2014). Progesterone elicits an inhibitory effect upon LPS-induced innate immune response in pre-labor human amniotic epithelium. *J. Reprod. Immunol.* 71 61–72. 10.1111/aji.12163 24128422

[B37] Fortunado S. Menon R. Bryant C. Lombardi S. (2000). Programmed cell death (apoptosis) as a possible pathway to metalloproteinase activation and fetal membrane degradation in premature rupture of membranes. *Am. J. Obstet. Gynecol.* 182 1468–1476. 10.1067/mob.2000.107330 10871467

[B38] Frevert C. W. Felgenhauer J. Wygrecka M. Nastase M. V. Schaefer L. J. (2018). Danger-associated molecular patterns derived from the extracellular matrix provide temporal control of innate immunity. *Histochem. Cytochem.* 66 213–227. 10.1369/0022155417740880 29290139 PMC5958376

[B39] Frey H. Schroeder N. Manon-Jensen T. Iozzo R. V. Schaefer L. (2013). Biological interplay between proteoglycans and their innate immune receptors in inflammation. *FEBS J.* 280 2165–2179. 10.1111/febs.12145 23350913 PMC3651745

[B40] Fulda S. Gorman A. M. Hori O. Samali A. (2010). Cellular stress responses: cell survival and cell death. *Int. J. Cell Biol.* 2010:214074. 10.1155/2010/214074 20182529 PMC2825543

[B41] Gaudet A. D. Popovich P. G. (2014). Extracellular matrix regulation of inflammation in the healthy and injured spinal cord. *Exp. Neurol.* 258 24–34. 10.1016/j.expneurol.2013.11.020 25017885 PMC4099942

[B42] Genbacev O. Vicovac L. Larcoque N. (2015). The role of chorionic cytotrophoblasts in the smooth chorion fusion with parietal decidua. *Placenta* 36 716–722. 10.1016/j.placenta.2015.05.002 26003500 PMC4476638

[B43] Ghavami S. Eshragi M. Ande S. R. Chazin W. J. Klonisch T. Halayko A. J. (2010). S100A8/A9 induces autophagy and apoptosis via ROS-mediated cross-talk between mitochondria and lysosomes that involves BNIP3. *Cell Res.* 20 314–331. 10.1038/cr.2009.129 19935772 PMC4161879

[B44] Gillaux C. Mehats C. Vaiman D. Cabrol D. Breuiller-Fouche M. (2011). Functional screening of TLRs in human amniotic epithelial cells. *J. Immunol.* 187 2766–2774. 10.4049/jimmunol.1100217 21775685

[B45] Goldenberg R. L. Culhane J. F. Iams J. D. Romero R. (2008). Epidemiology and causes of preterm birth. *Lancet* 371 75–84. 10.1016/S0140-6736(08)60074-4 18177778 PMC7134569

[B46] Gomez-Lopez N. Romero R. Panaitescu B. Leng Y. Xu Y. Tarca A. L. (2018). Inflammasome activation during spontaneous preterm labor with intra-amniotic infection or sterile intra-amniotic inflammation. *Am. J. Reprod. Immunol.* 80:e13049. 10.1111/aji.13049 30225853 PMC6419509

[B47] Gomez-Lopez N. Romero R. Plazyo O. Panaitescu B. Furcron A. E. Miller D. (2016). Intra-amniotic administration of HMGB1 induces spontaneous Preterm Labor and Birth. *Am. J. Reprod. Immunol.* 75 3–7. 10.1111/aji.12443 26781934 PMC5029853

[B48] Gravett M. Adams K. Sadowsky D. Grosvenor A. R. Witkin S. S. Axthelm M. K. (2007). Immunomodulator plus antibiotics delay preterm delivery after experimental intraamniotic infection in a nonhuman primate model. *Am. J. Obstet. Gynecol.* 197 518.e1–518.e8. 10.1016/j.ajog.2007.03.064 17980193 PMC2128777

[B49] Hadley E. E. Sheller-Miller S. Saade G. Salomon C. Mesiano S. Taylor R. N. (2018). Amnion epithelial cell-derived exosomes induce inflammatory changes in uterine cells. *Am. J. Obstet. Gynecol.* 219 478.e1–478.e21. 10.1016/j.ajog.2018.08.021 30138617 PMC6239974

[B50] Haruta M. Kishore A. H. Shi H. Keller P. W. Akgul Y. Word R. A. (2013). Fetal fibronectin signaling induces matrix metalloproteases and cyclooxygenase-2 (COX-2) in amnion cells and preterm birth in mice. *J. Biol. Chem.* 288 1953–1966. 10.1074/jbc.M112.424366 23184961 PMC3548503

[B51] Hasegawa-Nakamura K. Tateishi F. Nakamura T. Nakajima Y. Kawamata K. Douchi T. (2011). The possible mechanism of preterm birth associated with peridontopathic porphyromonas gingivalis. *J. Periodontal Res.* 46 497–504. 10.1111/j.1600-0765.2011.01366.x 21488875

[B52] Hayashi F. Smith K. D. Ozinsky A. Hawn T. R. Yi E. C. Goodlett D. R. (2001). The innate immune response to bacterial flagellin is mediated by Toll-like receptor 5. *Nature* 410 1099–1103. 10.1038/35074106 11323673

[B53] Hemmi H. Takeuchi O. Kawai T. Kaisho T. Sato S. Sanjo H. (2000). A Toll-like receptor recognizes bacterial DNA. *Nature* 408 740–745. 10.1038/35047123 11130078

[B54] Hirai A. H. Hayes D. K. Taualii M. M. Singh G. K. Fuddy L. J. (2013). Excess infant mortality among native hawaiians: identifying determinants for preventative action. *Am J Public Health.* 103 e88–e95. 10.2105/AJPH.2013.301294 24028241 PMC3828695

[B55] Hoang M. Potter J. A. Gysler S. M. Han C. S. Guller S. Norwitz E. R. (2014). Human fetal membranes generate distinct cytokine profiles in response to bacterial toll-like receptor and nod-like receptor agonists. *Biol. Reprod.* 90:39. 10.1095/biolreprod.113.115428 24429216 PMC4076407

[B56] Hsu C. Meaddough E. Bacherra H. Harirah H. Lu L. (2000). Increased apoptosis in human amnion is associated with labor at term. *Am. J. Reprod. Immunol.* 43 255–258. 10.1111/j.8755-8920-2000.430502.x 10872603

[B57] Huang H. Evanovich J. Yan W. Nace G. Zhang L. Ross M. (2011). Endogenous histones function as alarmins in sterile inflammatory liver injury, through Toll-like receptor 9 in mice. *Hepatology* 54 999–1008. 10.1002/hep.24501 21721026 PMC3213322

[B58] Hug H. Mohajeri M. H. La Fata G. (2018). Toll-like receptors: regulation of the immune response in the human gut. *Nutrients* 10:203. 10.3390/nu10020203 29438282 PMC5852779

[B59] Isnadi M. F. A. R. Chin V. K. Majid R. A. Lee T. Y. Abdullah M. A. Omenesa R. B. (2018). Critical Roles of IL-33/ST2 Pathway in Neurological Disorders. *Mediators Inflamm.* 2018:5346413. 10.1155/2018/5346413 29507527 PMC5817350

[B60] Jaiswal M. K. Agrawal V. Mallers T. Gilman-Sachs A. Hirsch E. Beaman K. D. (2013). Regulation of apoptosis and innate immune stimuli in inflammation-induced preterm labor. *J. Immunol.* 191 5702–5713. 10.4049/jimmunol.1301604 24163412 PMC3870276

[B61] Joyce E. M. Diaz P. Tamarkin S. Moore R. Strohl A. Stetzer B. (2016). In-vivo stretch of term human fetal membranes. *Placenta* 38 57–66. 10.1016/j.placenta.2015.12.011 26907383 PMC4768058

[B62] Kaga N. Katsuki Y. Obata M. Shibutani Y. (1996). Repeated administration of low-dose lipopolysaccharide induces preterm delivery in mice: a model for human preterm parturition and for assessment of the therapeutic ability of drugs against preterm delivery. *Am. J. Obstet. Gynecol.* 174 754–759. 10.1016/s0002-9378(96)70460-x 8623817

[B63] Karikó K. Ni H. Capodici J. Lamphier M. Weissman D. (2004). mRNA is an endogenous ligand for toll-like receptor 3. *J. Biol. Chem.* 279 12542–12550. 10.1074/jbc.M310175200 14729660

[B64] Katsura M. Ito A. Hirakawa S. Mori Y. (1989). Human recombinant interleukin-1alpha increases biosynthesis of collagenase and hyaluronic acid in cultured human chorionic cells. *FEBS Lett.* 224 315–318. 10.1016/0014-5793(89)80553-8 2537757

[B65] Kawai C. Kotani H. Miyao M. Ishida T. Jemail L. Abiru H. (2016). Circulating extracellular histones are clinically Relevant mediators of multiple organ injury. *Am. J. Pathol.* 186 829–843. 10.1016/j.ajpath.2015.11.025 26878212

[B66] Kawai T. Akira S. (2010). The role of pattern-recognition receptors in innate immunity: update on Toll-like receptors. *Nat. Immunol.* 11 373–384. 10.1038/ni.1863 20404851

[B67] Keelan J. A. Marvin K. W. Sato T. A. Coleman M. McCowan L. M. Mitchell M. D. (1999). Cytokine abundance in placental tissues: evidence of inflammatory activation in gestational membranes with term and preterm parturition. *Am. J. Obstet. Gynecol.* 181 1530–1536. 10.1016/s0002-9378(99)70400-x 10601939

[B68] Keith L. G. Oleszczuk J. J. (2002). Triplet births in the United States. An epidemic of high-risk pregnancies. *J. Reprod. Med.* 47 259–265. 12012876

[B69] Kendal-Wright C. E. (2007). Stretching, mechanotransduction and proinflammatory cytokines in the fetal membranes. *Reprod. Sci.* 14 35–41. 10.1177/1933719107310763 18089608

[B70] Kendal-Wright C. E. Hubbard D. Bryant-Greenwood G. D. (2008). Chronic stretching of amniotic epithelial cells increases pre-B cell colony-enhancing factor (PBEF/Visfatin) expression and protects them from apoptosis. *Placenta* 29 255–265. 10.1016/j.placenta.2007.12.008 18272217

[B71] Kendal-Wright C. E. Hubbard D. Gowin-Brown J. Bryant-Greenwood G. D. (2010). Stretch and inflammation-induced Pre-B cell colony-enhancing factor (PBEF/Visfatin) and Interleukin-8 in amniotic epithelial cells. *Placenta* 3 665–674. 10.1016/j.placenta.2010.06.007 20598369 PMC2921847

[B72] Koga K. Cardenas I. Aldo P. Abrahams V. M. Peng B. Fill S. (2009). Activation of TLR3 in the trophoblast is associated with preterm delivery. *Am. J. Reprod. Immunol.* 61 196–212. 10.1111/j.1600-0897.2008.00682.x 19239422 PMC2765929

[B73] Kumagai K. Otsuki Y. Ito Y. Shibata M. A. Abe H. Ueki M. (2001). Apoptosis in the normal human amnion at term, independent of Bcl-2 regulation and onset of labour. *Mol. Hum. Reprod.* 7 681–689. 10.1093/molehr/7.7.681 11420392

[B74] Kumar D. Fung W. Moore R. Pandey V. Fox J. Stetzer B. (2006). Proinflammatory cytokines found in amniotic fluid induce collagen remodeling, apoptosis and biophysical weakening of cultured human fetal membranes. *Biol. Reprod.* 74 29–34. 16148217 10.1095/biolreprod.105.045328

[B75] Kumar H. Kawai T. Akira S. (2009). Pathogen recognition in the innate immune response. *Biochem. J.* 420 1–16. 10.1042/BJ20090272 19382893

[B76] Lappas M. Odumetse T. Riley C. Reti N. G. Holdsworth-Carson S. J. Rice G. E. (2008). Pre-labor fetal membranes overlying the cervix display alterations in inflammation and NF-kappaB signaling pathways. *Placenta* 29 995–1002. 10.1016/j.placenta.2008.09.010 18952281

[B77] Leppert P. C. Takamoto N. Yu S. Y. (1996). Apoptosis in fetal membranes may predispose them to rupture. *J. Soc. Gynecol. Invest.* 3:128A.

[B78] Li L. Yang J. Jiang Y. Tu J. Schust D. J. (2015). Activation of decidual invariant natural killer T cells promotes lipopolysaccharide-induced preterm birth. *Mol. Hum. Reprod.* 21 369–381. 10.1093/molehr/gav001 25589517

[B79] Lim R. Barker G. Lappas M. (2018). Pellino 1 is a novel regulator of TNF and TLR signaling in human myometrial and amnion cells. *J. Reprod. Immunol.* 127 24–35. 10.1016/j.jri.2018.04.003 29751216

[B80] Lim R. Lappas M. (2015). A novel role for GSK3 in the regulation of the processes of human labour. *Reproduction* 149 189–202. 10.1530/REP-14-0493 25550525

[B81] Lim R. Lappas M. (2019). Expression and function of macrophage-inducible c-type lectin (mincle) in inflammation driven parturition in fetal membranes and myometrium. *Clin. Exp. Immunol.* 197 95–110. 10.1111/cei.13281 30793298 PMC6591154

[B82] Lim R. Tran H. T. Liong S. Barker G. Lappas M. (2016). The transcription factor interferon regulatory factor-1 (IRF1) plays a key role in the terminal effector pathways of human preterm labor. *Biol. Reprod.* 94:32. 10.1095/biolreprod.115.134726 26674566

[B83] Liong S. Lappas M. (2014). Endoplasmic reticulum stress is increased after spontaneous labor in human fetal membranes and myometrium where it regulates the expression of prolabor mediators. *Biol. Reprod.* 91:70. 10.1095/biolreprod.114.120741 25100709

[B84] Liu H. Redline R. W. Han Y. W. (2007). *Fusobacterium nucleatum* induces fetal death in mice via stimulation of TLR4-mediated placental inflammatory response. *J. Immunol.* 179 2501–2508. 10.4049/jimmunol.179.4.2501 17675512

[B85] Lund J. M. Alexopoulou L. Sato A. Karow M. Adams N. C. Gale N. W. (2004). Recognition of single-stranded RNA viruses by Toll-like receptor 7. *Proc. Natl. Acad. Sci. U.S.A.* 101 5598–5603. 10.1073/pnas.0400937101 15034168 PMC397437

[B86] Magna M. Pisetsky D. S. (2016). The alarmin properties of DNA and DNA-associated nuclear proteins. *Clin. Ther*. 38 1029–1041. 10.1016/j.clinthera.2016.02.029 27021604

[B87] Malak T. M. Bell S. C. (1994). Structural characteristics of term human fetal membranes: a novel zone of extreme morphological alteration within the rupture site. *Br. J. Obstet. Gynecol.* 101 375–386. 10.1111/j.1471-0528.1994.tb11908.x 8018607

[B88] Manabe Y. Yoshimura S. Mori T. Aso T. (1985). Plasma levels of 13,14-dihydro-15-keto prostaglandin F2 alpha, estrogens, and progesterone during stretch-induced labor at term. *Prostaglandins* 30 141–152. 4048477 10.1016/s0090-6980(85)80018-6

[B89] Maneta E. Warren A. Y. Hay D. P. Khan R. N. (2015). Caspase-1-mediated cytokine release from gestational tissues, placental and cord blood. *Front. Physiol.* 6:186. 10.3389/fphys.2015.00186 26157394 PMC4477139

[B90] Manuck T. A. (2017). Racial and ethnic differences in preterm birth. *Semin. Perinatol.* 41 511–518. 10.1053/j.semperi.2017.08.010 28941962 PMC6381592

[B91] McParland P. Taylor D. Bell S. (2003). Mapping of zoned of altered morphology and chorionic connective tissue phenotype in human fetal membranes (amniochorion and decidua) overlying the lower uterine pole and cervix before labor at term. *Am. J. Obstet. Gynecol.* 189 1481–1488. 14634589 10.1067/s0002-9378(03)00585-4

[B92] Meinert M. Eriksen G. V. Peterson A. C. Helmig R. B. Laurent C. Uldbjerg N. (2001). Proteoglycans and hyaluronan in fetal membranes. *Am. J. Obstet. Gynecol.* 184 679–685. 10.1067/mob.2001.110294 11262472

[B93] Meinert M. Malmstrom A. Tufvesson E. Westergren-Thorsson G. Peterson A. C. Laurent C. (2007). Labour induces increased concentrations of biglycan and hyaluronan in human fetal membranes. *Placenta* 28 482–486. 10.1016/j.placenta.2006.09.006 17125833

[B94] Menon R. (2019). Initiation of human parturition: signaling from senescent fetal tissues via extracellular vesicle mediated paracrine mechanism. *Obstet. Gynecol. Sci.* 62 199–211. 10.5468/ogs.2019.62.4.199 31338337 PMC6629986

[B95] Menon R. Bonney E. A. Condon J. Messiano S. Taylor R. N. (2016a). Novel concepts on pregnancy clocks and alarms: redundancy and synergy in human parturition. *Hum. Reprod. Update* 22 535–560. 10.1093/humupd/dmw022 27363410 PMC5001499

[B96] Menon R. Behnia F. Polettini J. Saade G. R. Campisi J. Velarde M. (2016b). Placental membrane aging and HMGB1 signaling associated with human parturition. *Aging* 8 216–230. 10.18632/aging.100891 26851389 PMC4789578

[B97] Menon R. Fortunato S. J. Yu J. Milne G. L. Sanchez S. Drobek C. O. (2011). Cigarette smoke induced oxidative stress and apoptosis in normal term fetal membranes. *Placenta* 32 317–322. 10.1016/j.placenta.2011.01.015 21367451

[B98] Menon R. Mesiano S. Taylor R. N. (2017). Programmed fetal membrane senescend and exosome-mediated signaling: a mechanism associated with timing of human parturition. *Front. Endocrinol.* 8:196. 10.3389/fendo.2017.00196 28861041 PMC5562683

[B99] Menon R. Richardson L. S. (2017). Preterm prelabor rupture of the membranes: a disease of the fetal membranes. *Semin. Perinatol.* 41 409–419. 10.1053/j.semperi.2017.07.012 28807394 PMC5659934

[B100] Merline R. Moreth K. Beckmann J. Nastase M. V. Zeng-Brouwers J. Tralhao J. G. (2011). Signaling by the matrix proteoglycan decorin controls inflammation and cancer through PDCD4 and microRNA-21. *Sci. Signal.* 4:ra75. 10.1126/scisignal.2001868 22087031 PMC5029092

[B101] Meylan E. Tschopp J. (2005). The RIP kinases: crucial integrators of cellular stress. *Trends Biochem. Sci.* 30 151–159. 10.1016/j.tibs.2005.01.003 15752987

[B102] Mi Y. Wang W. Zhang W. Liu C. Jiangwen L. Li W. (2017). Autophagic degradation of Collagen 1A1 by Cortisol in Human Amnion Fibroblasts. *Endocrinology* 158 1005–1014. 28323983 10.1210/en.2016-1829

[B103] Midwood K. Sacre S. Piccinini A. M. Inglis J. Trebaul A. Chan E. (2009). Tenascin-C is an endogenous activator of toll-like receptor 4 that is essential for maintaining inflammation in arthritic joint disease. *Nat. Med.* 15 774–780. 10.1038/nm 19561617

[B104] Millar L. K. Stollberg J. DeBuque L. Bryant-Greenwood G. (2000). Fetal membrane distension: determination of the intrauterine surface area and distention of the fetal membranes preterm and at term. *Am. J. Obstet. Gynecol.* 182 128–134. 10.1016/s0002-9378(00)70501-1 10649167

[B105] Miyake K. (2007). Innate immune sensing of pathogens and danger signals by cell surface Toll-like receptors. *Semin. Immunol.* 19 3–10. 10.1016/j.smim.2006.12.002 17275324

[B106] Moco N. P. Martin L. F. Pereira A. C. Polettini J. Peracoli J. C. Coelho K. I. (2013). Gene expression and protein localization of TLR-1, -2, -4, and -6 in amniochorion membranes of pregnancies complicated by histological chorioamnionitis. *Eur. J. Obstet. Gynecol. Reprod. Biol.* 171 12–17. 10.1016/j.ejogrb.2013.07.036 24125907

[B107] Mogami H. Hari Kishore A. Akgul Y. Word R. A. (2017). Healing of preterm ruptured fetal membranes. *Sci. Rep.* 7:13139. 10.1038/s41598-017-13296-1 29030612 PMC5640674

[B108] Motedayyen H. Fathi F. Fasihi-Ramandi M. Sabzghabaee A. M. Taheri R. A. (2019). Toll-like receptor 4 activation on human amniotic epithelial cells is a risk factor for pregnancy loss. *J. Res. Med. Sci.* 24:1 10.4103/jrms.JRMS_463_18 PMC6383334 30815014

[B109] Nadeau-Vallee M. Obari D. Palacois J. Brien M. E. Duval C. Chemtob S. (2016). Sterile Inflammation and pregnancy complications: a review. *Reproduction* 152 R277–R292. 10.1530/REP-16-0453 27679863

[B110] Nadeem L. Shynlova O. Matysiak-Zablocki E. Mesiano S. Dong X. Lye S. (2016). Molecular evidence of functional progesterone withdrawal in human myometrium. *Nat. Commun.* 7:11565. 10.1038/ncomms11565 27220952 PMC4894948

[B111] Nemeth E. Millar L. K. Bryant-Greenwood G. (2000). Fetal membrane distention: II. Differentially expressed genes regulated by acute distention *in vitro*. *Am. J. Obstet. Gynecol.* 182 60–67. 10.1016/s0002-9378(00)70491-1 10649157

[B112] Nie Y. Yang D. Oppenhiem J. J. (2016). Alarmins and antitumor immunity. *Clin. Ther.* 38 1042–1053. 10.1016/j.clinthera.2016.03.021 27101817 PMC6314656

[B113] Nizyaeva N. V. Kulikova G. V. Nagovitsyna M. N. Shchegolev A. I. (2019). Peculiarities of the expression of TLR4 and inhibitor of TLR-cascade tollip in the placenta in earlyand late-onset Preeclampsia. *Bull. Exp. Biol. Med.* 166 507–511. 10.1007/s10517-019-04383-6 30783843

[B114] Noll F. Behnke J. Leiting S. Trodi K. Alves G. T. Muller-Redetzky H. (2017). Self-extracellular RNA acts in synergy with exogenous danger signals to promote inflammation. *PLoS One* 12:e0190002. 10.1371/journal.pone.0190002 29261777 PMC5738100

[B115] Norman Ing N. Baker H. Ignacio V. Song C. Kendal-Wright C. E. (2019). “Stretch increases high-mobility group box 1 (HMGB1) secretion from human epithelial cells,” in *Proceedings of the 66th Annual Society for Reproductive Investigation Meeting* (Paris: Reproductive Biology F019).

[B116] Oosting M. Cheng S.-C. Bolscher J. M. Vestering-Stenger R. Plantinga T. S. Verschueren I. C. (2014). Human TLR10 is an anti-inflammatory pattern-recognition receptor. *Proc. Natl. Acad. Sci. U.S.A.* 111 E4478–E4484. 10.1073/pnas.1410293111 25288745 PMC4210319

[B117] Osman I. Young A. Jordon F. Greer I. A. Norman J. E. (2006). Leukocyte density and proinflammatory mediator expression in regional human fetal membranes and decidua before and during labor at term. *J. Soc. Gynecol. Investig.* 13 97–103. 10.1016/j.jsgi.2005.12.002 16443501

[B118] Park C. B. Braun K. L. Horiuchi B. Y. Tottori C. Onaka A. T. (2009). Longevity disparities in multiethnic Hawaii: an analysis of 2000 life tables. *Public Health Rep.* 124 579–584. 10.1177/003335490912400415 19618795 PMC2693172

[B119] Parry S. Strauss J. F. (1988). Premature rupture of the fetal membranes. *N. Engl. J. Med.* 338 663–670. 10.1056/NEJM199803053381006 9486996

[B120] Patel B. Peters G. A. Skomorovska-Prokvolit Y. Yi L. Tan H. Yousef A. (2018). Control of progesterone receptor-A transrepressive activity in myometrial cells: implications for the control of human parturition. *Reprod. Sci.* 25 214–221. 10.1177/1933719117716775 28671036 PMC6343215

[B121] Patkin E. C. Orr S. J. Schaible U. E. (2017). Macrophage inducible C-type Lectin as a multifunctional player in immunity. *Front. Immunol.* 8:861. 10.3389/fimmu.2017.00861 28791019 PMC5525440

[B122] Paulesu L. Bhattacharjee J. Bechi N. Romagnoli R. Jantra S. Ietta F. (2010). Pro-inflammatory cytokiens in animal and human gestation. *Curr. Pharm. Des.* 16 3601–3615. 10.2174/138161210793797933 20977424

[B123] Perales-Linares R. Navas-Martin S. (2013). Toll-like receptor 3 in viral pathogenesis: Friend or foe? *Immunology* 140 153–167. 10.1111/imm.12143 23909285 PMC3784162

[B124] Petes C. Odoardi N. Gee K. (2017). The toll for trafficking: toll-like receptor 7 delivery to the endosome. *Front. Immunol.* 8:1075. 10.3389/fimmu.2017.01075 28928743 PMC5591332

[B125] Phillippe M. (2014). Cell-free fetal DNA—a trigger for parturition. *N. Engl. J. Med.* 370 2534–2536. 10.1056/NEJMcibr1404324 24963574

[B126] Pirianov G. Waddington S. N. Lindstrom T. M. Terzidou V. Mehmet H. Bennett P. R. (2009). The cyclopentenone 15-deoxy-delta 12,14-prostaglandin J(2) delays lipopolysaccharide-induced preterm delivery and reduces mortality in the newborn mouse. *Endocrinology* 150 699–706. 10.1210/en.2008-1178 18845626

[B127] Plazyo O. Romero R. Unkel R. Balancio A. Mial T. N. Xu Y. (2016). HMGB1 induces an inflammatory response in the chorioamniotic membranes that is partially mediated by the inflammasome. *Biol. Reprod.* 95 1–14. 10.1095/biolreprod.116.144139 27806943 PMC5315428

[B128] Poltorak A. (1998). Defective LPS signaling in C3H/HeJ and C57BL/10ScCr mice: mutations in Tlr4 gene. *Science* 282 2085–2088. 10.1126/science.282.5396.2085 9851930

[B129] Poženel L. ALindenmair A. Schmidt K. Kozlov A. V. Grillari J. Wolbank S. (2019). Critical impact of human amniotic membrane tension on mitochondrial function and cell viability *in vitro*. *Cells* 2019:1641. 10.3390/cells8121641 31847452 PMC6953074

[B130] Qin S. Wang H. Yuan R. Li H. Ochani M. Ochani K. (2006). Role of HMGB1 in apoptosis-mediated sepsis lethality. *J. Exp. Med.* 203 1637–1642. 10.1084/jem.20052203 16818669 PMC2118346

[B131] Qiu X. Y. Sun L. Han X. L. Chang Y. Cheng L. Yin L. R. (2017). Alarmin high mobility group box-1 in maternal serum as a potential biomarker of chorioamnionitis-associated preterm birth. *Gynecol. Endocrinol.* 33 128–131. 10.1080/09513590.2016.1214260 27684473

[B132] Regan J. K. Kannan P. S. Kemp M. W. Kramer B. W. Newnham J. P. Jobe A. H. (2016). Damage-associated molecular pattern and fetal membrane vascular injury and collagen disorganization in lipopolysaccharide-induced intra-amniotic inflammation in fetal sheep. *Reprod. Sci.* 23 69–80. 10.1177/1933719115594014 26156854 PMC5933192

[B133] Relja B. Mors K. Marzi I. (2018). Danger signals in Trauma. *Eur. J. Trauma Emerg. Surg.* 44 301–316. 10.1007/s00068-018-0962-3 29728738 PMC6002466

[B134] Reti N. Lappas M. Riley C. Wlodek M. E. Permezel M. Walker S. (2007). Why do membranes rupture at term? Evidence of increased cellular apoptosis in the supracervical fetal membranes. *Am. J. Obstet. Gynecol.* 196 484.e1–484.e10. 10.1016/j.ajog.2007.01.021. 17466714

[B135] Richardson L. S. Taylor R. N. Menon R. (2020). Reversible EMT and MET mediate amnion remodeling during pregnancy and labor. *Sci. Signal.* 13:618. 10.1126/scisignal.aay1486 32047115 PMC7092701

[B136] Roedig H. Nastase M. V. Wygrecka M. Schaefer L. (2019). Breaking down chronic inflammatory diseases: the role of biglycan in promoting a switch between inflammation and autophagy. *FEBS J.* 286 2965–2979. 10.1111/febs.14791 30776184

[B137] Roh J. S. Sohn D. H. (2018). Damage-Associated molecular patterns in inflammatory disease. *Immune Netw.* 18:e27. 10.4110/in.2018.18.e27 30181915 PMC6117512

[B138] Romero R. Chaiworapongsa T. Alpay Savasan Z. A. Xu Y. Hussein Y. Dong Z. (2011). Damage-associated molecular patterns (DAMPs) in preterm labor with intact membranes and preterm PROM: a study of the alarmin HMGB1. *J. Matern. Fetal Neonatal Med.* 24 1444–1455. 10.3109/14767058.2011.591460 21958433 PMC3419589

[B139] Romero R. Espinoza J. Gonçalves L. F. Kusanovic J. P. Friel L. Hassan S. (2007). The role of inflammation and infection in preterm birth. *Semin. Reprod. Med.* 25 21–39. 10.1055/s-2006-956773 17205421 PMC8324073

[B140] Sato B. L. Collier E. S. Vermudez S. A. Junker A. D. Kendal-Wright C. E. (2016). Human amnion mesenchymal cells are pro-inflammatory when activated by the toll-like recetor 2/6 ligand, macrophage-activating lipoprotein-2. *Placenta* 44 69–79. 10.1016/j.placenta.2016.06.005 27452440 PMC4964608

[B141] Schaaf J. M. Liem S. M. S. Mol B. W. J. Abu-Hanna A. Ravelli A. C. J. (2013). Ethnic and racial disparities in the risk of preterm birth: a systematic review and meta-analysis. *Am. J. Perinatol.* 30 433–450. 23059494 10.1055/s-0032-1326988

[B142] Schaefer L. (2014). Complexity of danger: the diverse nature of damage-associated molecular patterns. *J. Biol. Chem.* 289 35237–35245. 10.1074/jbc.R114.619304 25391648 PMC4271212

[B143] Scharfe-Nugent A. Corr S. C. Carpenter S. B. Keogh L. Doyle B. Martin C. (2012). TLR9 provokes inflammation in response to fetal DNA: mechanism for fetal loss in preterm birth and preeclampsia. *J. Immunol.* 188 5706–5712. 10.4049/jimmunol.1103454 22544937

[B144] Scheibner K. A. Lutz M. A. Boodoo S. Fenton M. J. Powell J. D. Horton M. R. (2006). Hyaluronan fragments act as an endogenous danger signal by engaging TLR2. *J. Immunol.* 177 1272–1281. 10.4049/jimmunol.177.2.1272 16818787

[B145] Sheller-Miller S. Urrabaz-Garza R. Saade G. Menon R. (2017). Damage-associated molecular pattern markers HMGB1 and cell-free fetal telomere fragments in oxidative-stressed amnion epithelial cell-derived exosomes. *J. Reprod. Immunol.* 123 3–11. 10.1016/j.jri.2017.08.003 28858636 PMC5632595

[B146] Shen Z. Li E. Lu S. Shen J. Cai Y. M. Wu Y. E. (2008). Autophagic and apoptotic cell death in amniotic epithelial cells. *Placenta* 29 956–961. 10.1016/j.placenta.2008.09.001 18926571

[B147] Shim S. Romero R. Hong J. Park C. W. Jun J. K. Kim B. I. (2004). Clinical significance of intra-amniotic inflammation in patients with preterm premature rupture of membranes. *Am. J. Obstet. Gynecol.* 191 1339–1345. 10.1016/j.ajog.2004.06.085 15507963

[B148] Shynlova O. Tsui P. Jaffer S. Lye S. (2009). Integration of endocrine and mechanical signals in the regulation of myometrial functions during pregnancy and labour. *Eur. J. Obstet. Gynecol. Reprod. Biol.* 144 S2–S10. 10.1016/j.ejogrb.2009.02.044 19299064

[B149] Smith M. L. Gourdon D. Little W. C. Kubow K. E. Eguiluz R. A. Luna-Morris S. (2007). Force-induced unfolding of fibronectin in the extracellular matrix of living cells. *PLoS Biol.* 5:e268. 10.1371/journal.pbio.0050268 17914904 PMC1994993

[B150] So T. Ito A. Sato T. Mori Y. Hirakawa S. (1992). Tumor necrosis factor alpha stimulates the biosynthesis of matrix metalloproteinases and plasmogen activator in cultured human chorionic cells. *Biol. Reprod.* 46 772–778. 10.1095/biolreprod46.5.772 1317222

[B151] Son G. H. Kim Y. Lee J. J. Ham H. Song J. E. Park S. T. (2019). MicroRNA-548 regulates high mobility group box-1 expression in patients with preterm birth and chorioamnionitis. *Sci. Rep.* 9:19746. 10.1038/s41598-019-56327-9 31875024 PMC6930298

[B152] Stevens A. L. Wheeler C. A. Tannenbaum S. R. Grodzinsky A. J. (2008). Nitric oxide enhances aggrecan degradation by aggrecanase in response to TNF-α but not IL-1β treatment at a post-transcriptional level in bovine cartilage explants. *Osteoarthritis Cartilage* 16 489–497. 10.1016/j.joca.2007.07.015 17923423 PMC3263310

[B153] Strauss J. F. III (2013). Extracellular matrix dynamics and fetal membrane rupture. *Reprod. Sci.* 20 140–153. 10.1177/1933719111424454 22267536 PMC3826277

[B154] Strauss J. F. III Romero R. Gomez-Lopez N. Haymond-Thornburg H. Modi B. P. Teves M. E. (2018). Spontaneous preterm birth: advances toward the discovery of genetic predisposition. *Am. J. Obstet. Gynecol.* 218 294–314.e2. 10.1016/j.ajog.2017.12.009 29248470 PMC5834399

[B155] Taheri R. A. Motedayyen H. Ghotloo S. Masjedi M. Mosaffa N. Mirshafiey A. (2018). The effect of lipopolysaccharide on the expression level of immunomodulatory and immunostimulatory factors of human amniotic epithelial cells. *BMC Res. Notes* 11:343. 10.1186/s13104-018-3411-9 29843819 PMC5975661

[B156] Takeda K. (2004). Toll-like receptors in innate immunity. *Int. Immunol.* 17 1–14. 10.1093/intimm/dxh186 15585605

[B157] Takeda K. Kaisho T. Akira S. (2003). Toll-like receptors. *Annu. Rev. Immunol.* 21 335–376. 10.1146/annurev.immunol.21.120601.141126 12524386

[B158] Takeuchi O. Akira S. (2010). Pattern recognition receptors and inflammation. *Cell* 140 805–820. 10.1016/j.cell.2010.01.022 20303872

[B159] Takeuchi O. Hoshino K. Kawai T. Sanjo H. Takada H. Ogawa T. (1999). Differential roles of TLR2 and TLR4 in recognition of gram-negative and gram-positive bacterial cell wall components. *Immunity* 11 443–451. 10.1016/s1074-7613(00)80119-3 10549626

[B160] Takeuchi O. Kawai T. Mühlradt P. F. Morr M. Radolf J. D. Zychlinsky A. (2001). Discrimination of bacterial lipoproteins by Toll-like receptor 6. *Int. Immunol.* 13 933–940. 10.1093/intimm/13.7.933 11431423

[B161] Takeuchi O. Sato S. Horiuchi T. Hoshino K. Takeda K. Dong Z. (2002). Cutting edge: role of toll-like receptor 1 in mediating immune response to microbial lipoproteins. *J. Immunol.* 169 10–14. 10.4049/jimmunol.169.1.10 12077222

[B162] Tateishi F. Hasegawa-Nakamura K. Nakamura T. Oogai Y. Komatsuzawa H. Kawamata K. (2012). Detection of *fusobacterium nucleatum* in chorionic tissues of high-risk pregnant women. *J. Clin. Periodontal.* 39 417–424. 10.1111/j.1600-051X.2012.01855.x 22304677

[B163] Than G. N. Romero R. Tarca A. L. Draghici T. S. Erez O. Chaiworapongsa T. (2009). Mitochondrial manganese superoxide dismutase mRNA expression in human chorioamniotic membranes and its association with labor, inflammation and infection. *J. Matern. Fetal Neonatal Med.* 22 1000–1013. 10.3109/14767050903019676 19900038 PMC2823267

[B164] Thaxton J. E. Nevers T. A. Sharma S. (2010). TLR-mediated preterm birth in response to pathogenic agents. *Infect. Dis. Obstet. Gynecol.* 2010:378472. 10.1155/2010/378472 20827416 PMC2933901

[B165] Thompson M. R. Kaminski J. J. Kurt-Jones E. A. Fitzgerald K. A. (2011). Pattern recognition receptors and the innate immune response to viral infection. *Viruses* 3 920–940. 10.3390/v3060920 21994762 PMC3186011

[B166] Tolle L. B. Standiford T. J. (2013). Danger-associated molecular patterns (DAMPs) in acute lung injury. *J. Pathol.* 229 145–156. 10.1002/path.4124 23097158

[B167] Tsark J. Braun K. L. J. (2009). Eyes on the Pacific: cancer Issues of Native Hawaiians and Pacific Islanders in Hawai’i and the US-Associated Pacific. *J. Cancer Educ.* 24 S68–S69. 10.1080/08858190903404619 20024833 PMC2914228

[B168] Venereau E. Ceriotti C. Bianchi M. E. (2015). DAMPs from cell death to new life. *Front. Immunol.* 18:422. 10.3389/fimmu.2015.00422 26347745 PMC4539554

[B169] Waldorf A. K. M. Persing D. Novy M. J. Sadowsky D. W. Gravett M. G. (2008). Pretreatment with toll-like receptor 4 antagonist inhibits lipopolysaccharide-induced preterm uterine contractility, cytokines, and prostaglandins in rhesus monkeys. *Reprod. Sci.* 15 121–127. 10.1177/1933719107310992 18187405 PMC2774271

[B170] Waldorf A. K. M. Singh N. Mohan A. R. Young R. C. Ngo L. Das A. (2015). Uterine overdistention induces preterm labor mediated by inflammation: observations in pregnant women and nonhuman primates. *Am. J. Obstet. Gynecol.* 213 830.e1–830.e19. 10.1016/j.ajog.2015.08.028 26284599 PMC4679421

[B171] Wallin R. P. A. Lundqvist A. Moré S. H. von Bonin A. Kiessling R. Ljunggren H.-G. (2002). Heat-shock proteins as activators of the innate immune system. *Trends Immunol.* 23 130–135. 10.1016/s1471-4906(01)02168-8 11864840

[B172] Wang H. Hirsch E. (2003). Bacterially-induced preterm labor and regulation of prostaglandin-metabolizing enzyme expression in mice: the role of toll-like receptor 4. *Biol. Reprod.* 69 1957–1963. 10.1095/biolreprod.103.019620 12904319

[B173] Wang S. Cao C. Piao H. Li Y. Tao Y. Zhang X. (2015). Tim-3 protects decidual stromal cells from toll-like receptorr-mediated apoptosis and inflammatory reactions and promotes Th2 bias at the maternal-fetal interface. *Sci. Rep.* 5:9013. 10.1038/srep09013 25757669 PMC4355741

[B174] Wang Y. W. Wang W. S. Wang L. Y. Bao Y. R. Lu J. W. Lu Y. (2019). Extracellular matrix remodeling effects of serum amyloid A1 in the amnion: implications for fetal membrane rupture. *Am. J. Reprod. Immunol.* 81:e13073. 10.1111/aji.13073 30461130

[B175] Waring G. J. Robson S. C. Bulmer J. N. Tyson-Capper A. J. (2015). Inflammatory signaling in fetal membranes: increased expression levels of TLR1 in the presence of preterm histological chorioamnionitis. *PLoS One* 10:e0124298. 10.1371/journal.pone.0124298 25965269 PMC4429010

[B176] West A. P. Koblansky A. A. Ghosh S. (2006). Recognition and signaling by toll-like receptors. *Annu. Rev. Cell Dev. Biol.* 22 409–437. 10.1146/annurev.cellbio.21.122303.115827 16822173

[B177] Wight T. N. Kang I. Merrilees M. J. (2014). Versican and the control of inflammation. *Matrix Biol.* 35 152–161. 10.1016/j.matbio.2014.01.01 24513039 PMC4039577

[B178] Wong Y. P. Tan G. C. Wong K. K. Anushia S. Cheah F. C. (2018). Gardnerella vaginalis in perinatology: an overview of the clinicopathological correlation. *Malays J. Pathol.* 40 267–286. 30580358

[B179] Xia C. Braunstein Z. Toomey A. C. Zhong J. Rao X. (2018). S100 proteins as a important regulator of macrophage inflammation. *Front. Immunol.* 8:1908. 10.3389/fimmu.2017.01908 29379499 PMC5770888

[B180] Xu D. Young J. Song D. Esko J. D. (2011). Heparan sulfate is essential for high mobility group protein 1 (HMGB1) receptor signaling by the receptor for advanced glycation end products (RAGE). *J. Biol. Chem.* 286 41736–41744. 10.1074/jbc.M111.299685 21990362 PMC3308882

[B181] Yang H. Wang H. Czura C. J. Tracey K. J. (2005). The cytokine activity of HMGB1. *J. Leukoc. Biol.* 78 1–8. 15734795 10.1189/jlb.1104648

[B182] Zaga V. Estrada-Gutierrez G. Beltran-Montoya J. Maida-Claros R. Lopez-Vancell R. Vadillo-Ortega F. (2004). Secretions of interleukin-1beta and tumor necrosis factor alpha by whole fetal membranes depend on initial interactions of amnion or choriodecidua with lipopolysaccharide or group B Streptococci. *Biol. Rep.* 71 1296–1302. 10.1095/bioreprod.104.028621 15201199

[B183] Zhang J. Z. Liu Z. Liu J. Ren J. X. Sun T. S. (2014). Mitochondrial DNA induces inflammation and increases TLR9/NF-κB expression in lung tissue. *Int. J. Mol. Med.* 33 817–824. 10.3892/ijmm.2014.1650 24535292 PMC3976143

[B184] Zhang Q. Kiyoshi I. Carl H. J. (2010). Mitochondrial dna is released by shock and activates neutrophils via p38 map kinase. *Shock.* 34 55–59. 10.1097/SHK.0b013e3181cd8c08 19997055

[B185] Zhou H. Yu M. Zhao J. Martin B. N. Roychowdhury S. McMullen M. R. (2016). IRAKM-Mincle axis links cell death to inflammation: pathophysiological implications for chronic alcoholic liver disease. *Hepatology* 64 1978–1993. 10.1002/hep.28811 27628766 PMC5115953

